# The role of gut microbiota in the occurrence and progression of non-alcoholic fatty liver disease

**DOI:** 10.3389/fmicb.2023.1257903

**Published:** 2024-01-05

**Authors:** Huanzhuo Mai, Xing Yang, Yulan Xie, Jie Zhou, Qing Wang, Yiru Wei, Yuecong Yang, Dongjia Lu, Li Ye, Ping Cui, Hao Liang, Jiegang Huang

**Affiliations:** ^1^School of Public Health, Guangxi Medical University, Nanning, China; ^2^Guangxi Key Laboratory of AIDS Prevention and Treatment, Guangxi Medical University, Nanning, China; ^3^Joint Laboratory for Emerging Infectious Diseases in China (Guangxi)-ASEAN, Nanning, China; ^4^Life Sciences Institute, Guangxi Medical University, Nanning, China; ^5^Guangxi Colleges and Universities Key Laboratory of Prevention and Control of Highly Prevalent Diseases, Guangxi Medical University, Nanning, China

**Keywords:** gut microbiota, 16S ribosomal RNA gene amplicon sequencing, nonalcoholic fatty liver disease, liver disease, microbial markers

## Abstract

**Background:**

Non-alcoholic fatty liver disease (NAFLD) is the most prevalent cause of chronic liver disease worldwide, and gut microbes are associated with the development and progression of NAFLD. Despite numerous studies exploring the changes in gut microbes associated with NAFLD, there was no consistent pattern of changes.

**Method:**

We retrieved studies on the human fecal microbiota sequenced by 16S rRNA gene amplification associated with NAFLD from the NCBI database up to April 2023, and re-analyzed them using bioinformatic methods.

**Results:**

We finally screened 12 relevant studies related to NAFLD, which included a total of 1,189 study subjects (NAFLD, *n* = 654; healthy control, *n* = 398; obesity, *n* = 137). Our results revealed a significant decrease in gut microbial diversity with the occurrence and progression of NAFLD (SMD = −0.32; 95% CI −0.42 to −0.21; *p* < 0.001). Alpha diversity and the increased abundance of several crucial genera, including *Desulfovibrio*, *Negativibacillus*, and *Prevotella*, can serve as an indication of their predictive risk ability for the occurrence and progression of NAFLD (all AUC > 0.7). The occurrence and progression of NAFLD are significantly associated with higher levels of LPS biosynthesis, tryptophan metabolism, glutathione metabolism, and lipid metabolism.

**Conclusion:**

This study elucidated gut microbes relevance to disease development and identified potential risk-associated microbes and functional pathways associated with NAFLD occurrence and progression.

## 1 Introduction

Non-alcoholic fatty liver disease (NAFLD) is metabolic stress–induced liver damage characterized by excess triglyceride accumulation in the liver without consumption of excessive alcohol (Iizuka et al., 2018; Jiang et al., 2022). It is the dominant cause of chronic liver disease worldwide, with a prevalence of approximately 25% (Lu et al., 2021). NAFLD encompasses a spectrum ranging from non-alcoholic simple fatty liver (NAFL) to non-alcoholic steatohepatitis (NASH), then progress to liver cirrhosis, fibrosis, and hepatocellular carcinoma (HCC) (Li et al., 2023). Although NAFLD carries a relatively benign prognosis and is mostly asymptomatic, up to 50% of affected individuals will develop NASH, cirrhosis, and liver fibrosis (Sunny et al., 2011; Zhang T. et al., 2019). Patients may potentially develop liver cancer and bladder cancer, due to the mediation of insulin resistance (Tarantino et al., 2021), which could ultimately increase liver-related morbidity and mortality (Abdul-Hai et al., 2015). However, the pathogenesis of NAFLD, specifically the disease progression to NASH, remains incompletely understood. This information is crucial to reverse and prevent the occurrence and development of the disease (Navarro et al., 2015). To date, liver biopsy is the gold standard for the diagnosis of NAFLD, but it cannot be applied at a large scale due to its associated risk (Zhang et al., 2023). Hence, accurate and precise non-invasive biomarkers are needed to detect NAFLD and its progression.

Accumulating evidence has shown that the gut microbiome is involved in the pathogenesis and progression of NAFLD (Boursier et al., 2016; Ponziani et al., 2019; Saeedi et al., 2020; Zhang et al., 2021). Consistently, gut microbial dysbiosis appears to modify susceptibility to NAFLD. Lee et al. (2020) found a significant decrease in the diversity of microbiota in patients with biopsy-proven NAFLD compared with non-NAFLD controls. In contrast, Jiang et al. (2015) reported no significant difference in the microbiota biodiversity between patients with NAFLD and healthy controls in their study. Lang et al. (2021) found that the degree of inflammation and stages of fibrosis are associated with low abundances of *Faecalibacterium*, *Bacteroides*, and *Prevotella*. And Boursier et al. (2016) reported that the abundance of *Bacteroides* is significantly increased in patients with NASH, whereas the abundance of *Prevotella* is decreased. The results imply that gut flora characteristics may vary across different stages of NAFLD. However, it is worth noting that these studies were conducted exclusively in European regions, and due to the strong correlation between ethnicity and the gut microbiota (Human Microbiome Project Consortium, 2012), the composition of the intestinal flora in non-European populations may differ significantly. The exact composition of the gut microbiota in patients with NAFLD, as well as the alterations in the gut microbiota composition across early and advanced stages of the disease, are currently unknown.

In the present study, we obtained 16S ribosomal RNA (rRNA) gene amplicon sequencing data associated with the gut microbiota from publicly available databases for all patients with NAFLD who met our screening criteria. We submitted the data to a rigorous biostatistical analysis to explore changes in the diversity and composition of the gut microbiota in patients with NAFLD as the disease progresses, and to identify the key microbial and functional pathways associated with NAFLD.We aimed to elucidate the relationship between alterations in the intestinal microbiome and the occurrence and development of NAFLD and to provide references for future screening of non-invasive biomarkers for the NAFLD diagnosis.

## 2 Materials and methods

### 2.1 Search strategy and selection criteria

We identified studies of human fecal flora associated with NAFLD by 16S rRNA gene sequencing up to April 2023 from the National Center for Biotechnology Information (NCBI) database (PubMed^1^ and BioProject.^2^) We developed out search strategy based on keywords, medical subject headings (MeSH) terms, and synonyms (Supplementary Table S1). We selected studies based on the following criteria: (1) The study groups contained patients with NAFLD and controls. The controls included healthy controls and patients with obesity but not NAFLD. The diagnostic methods and criteria for each study regarding the NAFLD status and the control group are shown in Supplementary Table S2. “Pre-progression” indicates the pre-progressive state of NAFLD-related diseases, and “post-progression” indicates the post-progressive state of NAFLD-related diseases. (2) The sample type was fecal samples or rectal swabs. (3) We excluded randomized controlled trials. (4) The sample sequencing method was 16S rRNA gene amplicon sequencing and the sequencing data were publicly available. We obtained raw 16S rRNA gene sequences and associated metadata from publicly available databases or directly from the authors. We excluded studies involving animal experiments or *in vitro* investigations, as well as reviews, meta-analyses, comments, letters, poster abstracts, and those with a sample size of fewer than three individuals in the case or control groups.

### 2.2 Processing of raw data

We download the Sequence Read Archive (SRA) files from NCBI and converted them to raw FASTQ files by using fastq-dump in the SRA Toolkit.^3^ We imported these FASTQ files into Quantitative Insights in Microbial Ecology (QIIME) version 2–2021.2 for processing and bioinformatics analysis.^4^ We processed the raw sequences to remove primers by using “q2-cutadapt.”^5^ We trimmed primer sequences, chimeras, and low-quality read ends with a quality score below 35 by using the Divisive Amplicon Denoising Algorithm 2 (DADA2) plugin for QIIME 2. The reads were trimmed using parameters in the DADA2 plugin carried out for each study, which can be found in Supplementary Table S3. We used DADA2 (Callahan et al., 2016), implemented in QIIME2, to model errors, to filter the raw FASTQ files, and to remove chimeras. After DADA2 denoising to generate a table of amplicon sequences variants (ASVs) and representative sequences, we filtered samples with a frequency of less than 10 in the feature table by using the “q2-feature-table filter-features” so that low-quality samples did not affect the results of downstream analysis. We annotated the species for each representative ASV by using a pre-trained plain Bayesian classifier (Bokulich et al., 2018) based on the latest version of the SILVA 138 reference database (clustered at 99% similarity). To minimize the effects of different sequencing platforms, sequencing regions, and primer amplifications, we combined the feature tables and representative sequences generated from each separate research by using the “qiime feature-table merge” and “qiime feature-table merge-seqs” commands in QIIME 2. We constructed a reliable phylogenetic tree by inserting the sequences into the SILVA 128 reference tree using the q2-fragment-insertion plugin, through the SATé-enabled phylogenetic placement (SEPP) algorithm, which is commonly used for meta-analyses of microbiome data (Janssen et al., 2018). For the merged feature table, we employed normalization methods to process the large disparity in the sequencing depths of different studies: We rarefied samples to 2,000 and converted them to a centered log-ratio (CLR) with the R package mecodev (Liu et al., 2021).

### 2.3 Data analysis

We imported files of the filtered ASV feature table, phylogenetic tree, metadata, representative sequences, and taxonomic classifications into RStudio 4.2.1 (RStudio, Inc., Boston, MA, USA). Using the R package microeco (Liu et al., 2021), we eliminated ASVs with taxonomic assignments labeled as “mitochondria” or “chloroplast” and created an object of microtable class for subsequent analysis. To mitigate the impact of sequencing depth on diversity measurements, we performed random re-sampling of all samples in the ASV abundance matrix based on the lowest sequencing depth andnormalized the ASV feature table. We employed a sampling-based ASV analysis and used the R package vegan to calculate the alpha diversity indices, including richness (observed species, Chao1, and abundance-based coverage estimator [ACE]), diversity (Shannon, Simpson, Invsimpson, and Fisher), and Faith’s phylogenetic diversity (PD) to evaluate the overall structure of the gut microbiota.^6^ We determined beta diversity within and between studies with principal coordinates analysis (PCoA) based on the unweighted UniFrac distance and the weighted UniFrac distance, and we employed permutational multivariate analysis of variance (PERMANOVA) with 999 replications on each distance metric. Meanwhile, we calculated bacterial beta diversity with PCoA based on the Bray–Curtis distance to show the species diversity for each study. Besides, based on the distribution of ASVs, we constructed a Venn diagram to show the ASV intersections among different groups through the R package VennDiagram.^7^

We performed bacterial taxonomic analyses and comparisons between groups based on the level of bacterial phylum, class, order, family, and genus with the Wilcoxon rank-sum test. We applied the linear discriminant analysis effect size (LEfSe) method (Segata et al., 2011) to analyze fecal microbial characterization between cases and controls, and we evaluated the effect size of each feature by linear discriminant analysis (LDA, with an LDA score [log10] = 3 as the cut-off value).

We used the R package Tax4Fun2 (Wemheuer et al., 2020) to predict the Kyoto Encyclopedia of Genes and Genomes (KEGG) functional pathways related to the microbial community based on 16S rRNA gene sequencing data classified on the SILVA 99Ref database. We compared between groups based on with functional abundance of bacterial communities to obtain functional pathways that differed significantly between the groups and visualized enrichment through the R package ggplot2.^8^ Ultimately, we used the area under the curve (AUC) of the receiver operating characteristic (ROC) curve to evaluate the predictive effectiveness of the alpha diversity indices and differential microbial taxonomy.

We could divide patients with NAFLD into the pre-progressive period or the post-progressive period. The pre-progressive period of NAFLD includes NAFL and NAFLD (assuming the study is a subgroup of NAFLD progress). The post-progressive period of NAFLD includes NASH, NAFLD fibrosis, and NAFLD cirrhosis. We compared gut microbial diversity, the abundance of bacterial taxonomy, and KEGG functional pathways between patients with NAFLD and healthy controls, patients with NAFLD and patients with obesity, or between the pre-progressive and post-progressive periods of NAFLD. We used the R package meta to generate the forest plots of the comparisons of the alpha diversity.^9^ We explored heterogeneity by calculating I^2^ and interpreted it as follows: < 25% indicates low heterogeneity, 25–45% indicates moderate heterogeneity, and > 45% indicates significant heterogeneity. If there was significant heterogeneity between studies (I^2^ > 45%), we applied the random-effect model; otherwise, we applied the fixed-effects model. We used the standardized mean difference (SMD) and the corresponding 95% credible interval (CI) evaluate the results. We conducted ROC curve analysis with GraphPad Prism 9.4 (GraphPad Software, Inc., San Diego, CA, USA). We considered a model with an AUC of >0.7 to be acceptable or better. All tests were two-sided with a value of *p* of 0.05 set as the threshold for significance.

## 3 Results

### 3.1 Study characteristics

**Figure 1:**
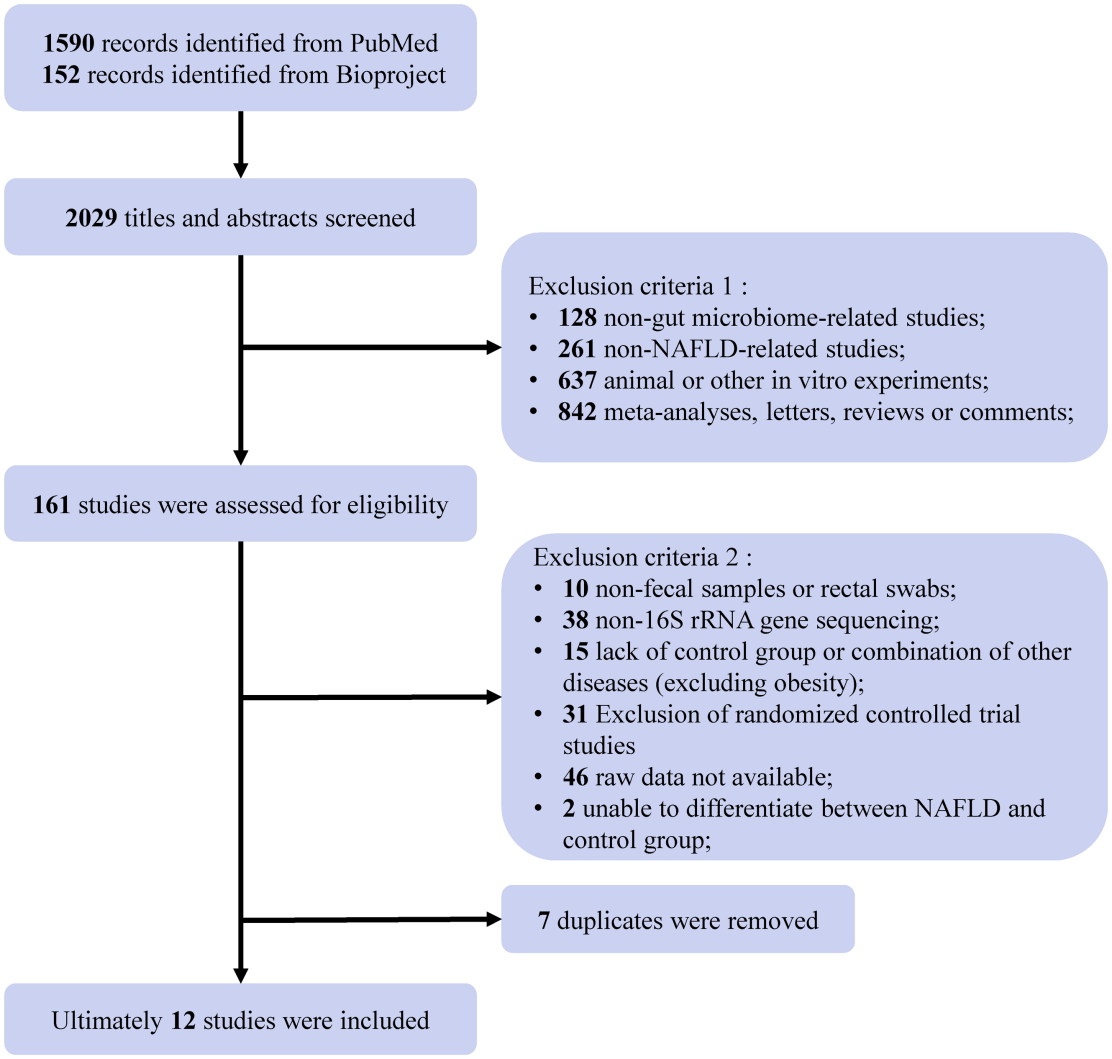
Flow chart of the selection procedure. A comprehensive search of the NCBI databases (PubMed and Bioproject) up to April 2023 yielded 2029 records related to NAFLD. After eliminating non-intestinal flora studies, non-NAFLD-related studies, animal or *in vitro* experiments, meta-analyses, letters, reviews, or comments, a total of 161 studies or projects were assessed for eligibility. Subsequently, further screening was conducted to exclude non-fecal samples or non-rectal swabs, non-16S rRNA gene sequencing, lacking controls, raw data not available, not distinguishing between NAFLD and control, and duplicates. Ultimately, the inclusion of 12 studies for analysis.

We found a total of 1,590 items from PubMed and 152 items from BioProject based on the search criteria, among which we included 12 NAFLD-related studies for subsequent analysis (Jiang et al., 2015; Wang et al., 2017; Caussy et al., 2019; Zhang Y. et al., 2019; Dong et al., 2020; Monga Kravetz et al., 2020; Ahmed et al., 2021; Baumann et al., 2021; Kordy et al., 2021; Lang et al., 2021; Pan et al., 2021; Liang et al., 2022) (Figure 1). Most participants included in the studies were diagnosed with NAFLD, while four of the included studies (Caussy et al., 2019; Dong et al., 2020; Kordy et al., 2021; Pan et al., 2021) included participants diagnosed with NAFL, NASH, and liver cirrhosis or fibrosis associated with NAFLD. These 12 studies were conducted in five countries, including five from China, four from the United States, two from Europe (one from Germany and one from Austria), and one from Canada. There were containing a total of 1,189 individual samples (NAFLD, *n* = 654; healthy control, *n* = 398; obesity, *n* = 137). Table 1 provides comprehensive details of the characteristics of included studies.

**Table 1:** Study characteristics of the included studies.

Study	PMID	BioProject accession number	Country	Study period	Sample size	16S rRNA Variable
Monga Kravetz et al., 2020	32561908	PRJNA328258 PRJNA606577	the United States	NA	NAFLD = 45, Obesity = 30	V4, Illumina MiSeq, Paired-end sequencing
Pan et al., 2021	34778099	PRJNA737039	China	2019.06–2019.12	NAFL = 25, NASH = 25, Obesity = 25	V4,Illumina HiSeq 1,500, Paired-end sequencing
Jiang et al., 2015	25644696	PRJNA246121	China	2013.05–2013.10	NAFLD = 53, Healthy Control = 32	V3,Illumina HiSeq 2000, Paired-end sequencing
Dong et al., 2020	32066758	PRJNA542724	the United States	2017.06.-2018.06	NAFLD (no fibrosis) = 16, NAFLD (advanced fibrosis) = 7, Healthy Control = 25	V4, HiSeq 2,500, Paired-end sequencing
Lang et al., 2021	33896117	PRJNA540738	Germany	2015.03–2018.12	NAFLD = 131, Healthy control = 19	V3,Ion Torrent S5, Single read sequencing
Caussy et al., 2019	30926798	PRJEB28350	the United States	2011.12–2017.12	NAFLD = 51, NAFLD-CIR = 26, Healthy Control = 117	V4, Illumina MiSeq, Single read sequencing
Zhang Y. et al., 2019	31726978	PRJNA541489	China	2018.01–2018.09	NAFLD = 24, Healthy Control = 23	V3-V4,Illumina HiSeq 2000 Paired-end sequencing
Ahmed et al., 2021	34622234	PRJNA682382	Canada	2016.06–2018.03	NAFLD = 29, Healthy Control = 30	V3-V4,Illumina MiSeq, Paired-end sequencing
Baumann et al., 2021	34497333	PRJEB41058	Austria	NA	NAFLD = 21, Healthy Control = 9	V1-V2,Illumina MiSeq, Paired-end sequencing
Wang et al., 2017	29180991	PRJNA382861	China	NA	NAFLD = 31, Healthy Control = 26	V3-V4, Illumina MiSeq, Paired-end sequencing
Liang et al., 2022	36364902	PRJCA010192	China	NA	NAFLD = 63, Healthy Control = 63	V3-V4,Illumina Hiseq 2,500, Paired-end sequencing
Kordy et al., 2021	34475864	PRJNA480711	the United States	2016.09–2017.06	NASH = 20, NAFL = 87, Healthy Control = 54, Obesity = 82	V4,Illumina MiSeq, Paired-end sequencing

### 3.2 Gut microbial diversity decreases significantly with the onset and progression of NAFLD

**Figure 2:**
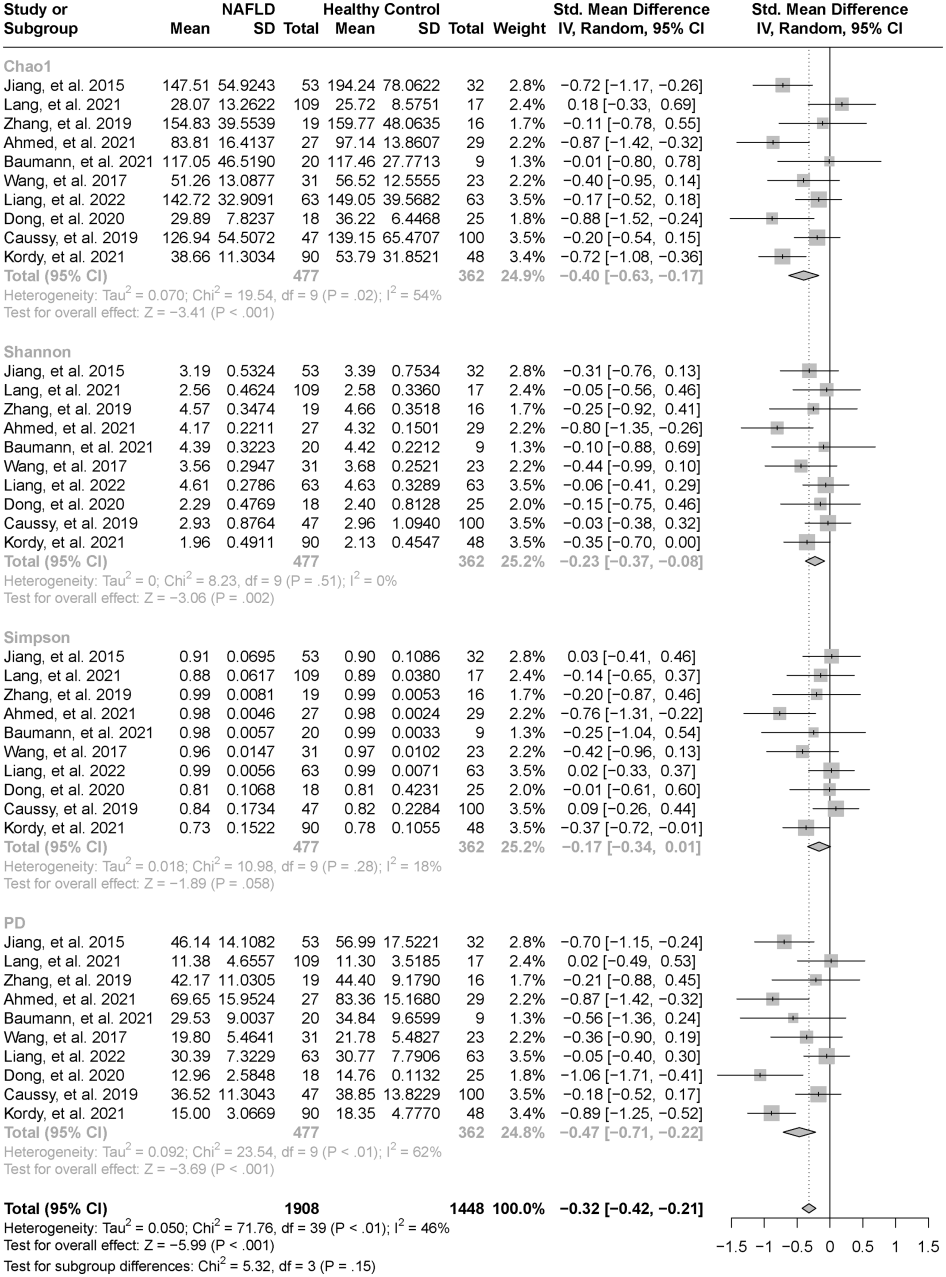
The forest plot shows the outcomes of a meta-analysis performed on the alpha-diversity of each parameter in both the NAFLD and healthy control groups. As estimated by the Chao1, Shannon, and PD indexes, the gut microbial alpha diversity was reduced significantly in NAFLD patients, compared with healthy controls. The pooled results showed a significant decrease in alpha diversity in NAFLD compared to the healthy control group.

**Figure 3:**
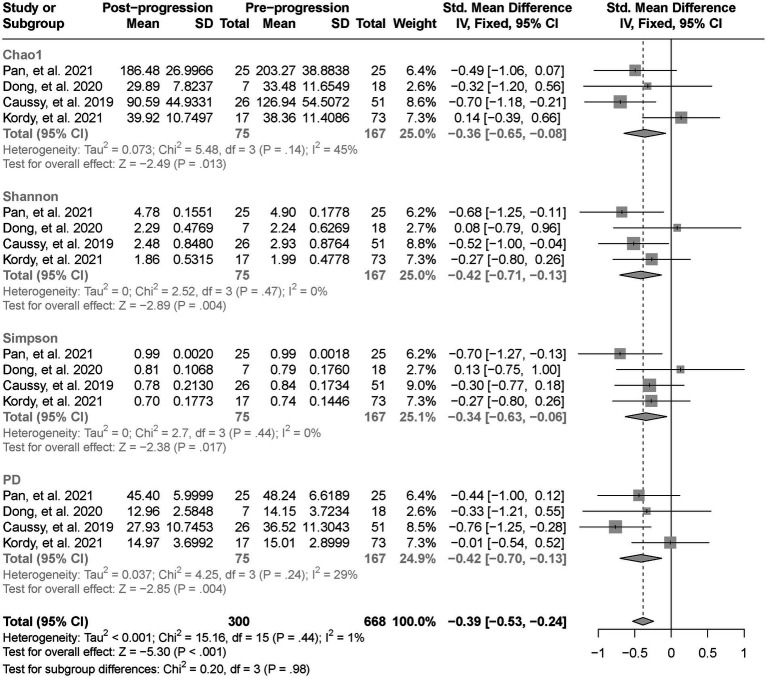
The forest plot shows the outcomes of a meta-analysis performed on the alpha-diversity of each parameter in both the post-NAFLD and pre-NAFLD groups. The pooled results showed a significant decrease in alpha diversity in post-progressive period of NAFLD compared to the post-progressive period of NAFLD.

**Figure 4:**
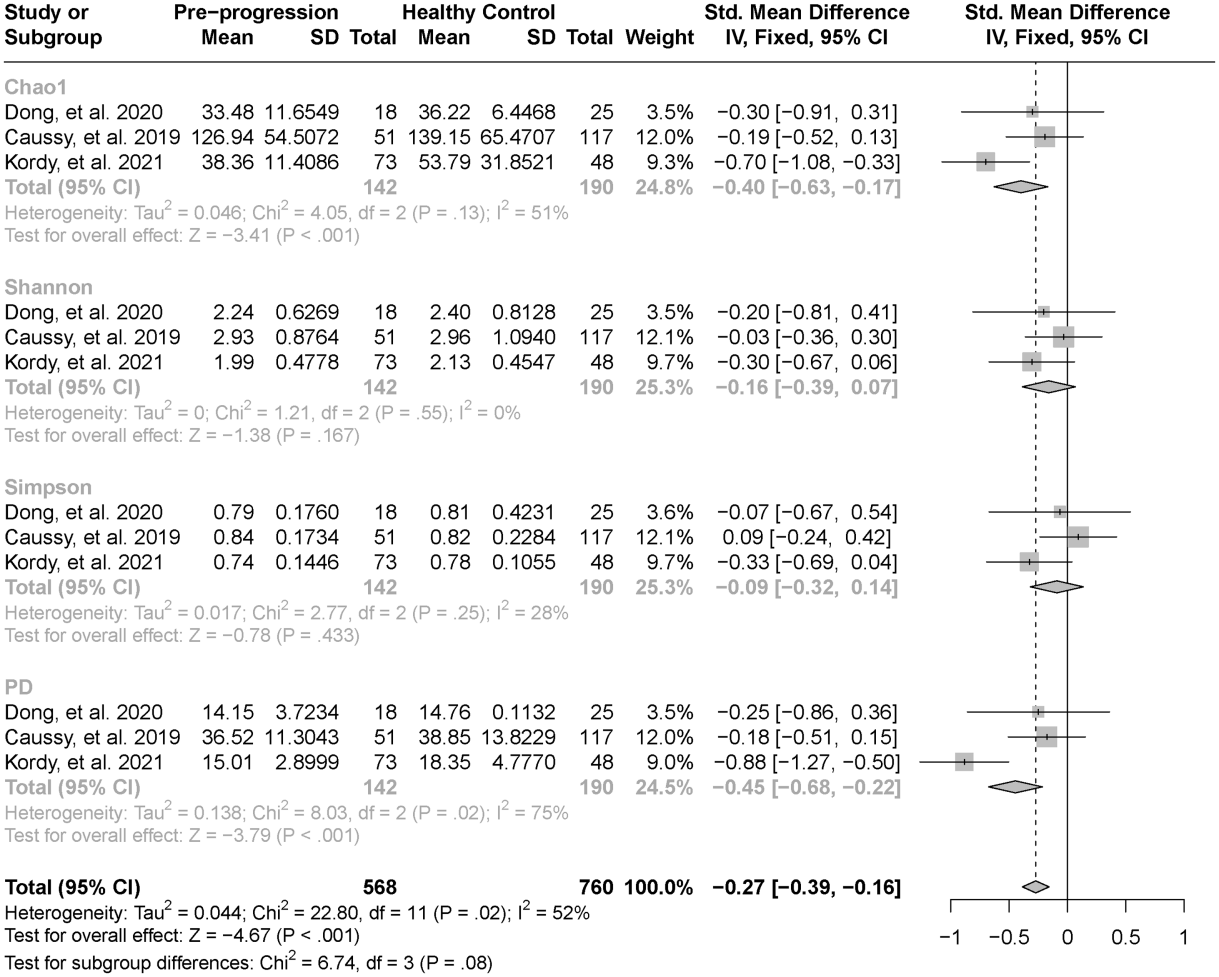
The forest plot shows the outcomes of a meta-analysis performed on the alpha-diversity of each parameter in both the post-NAFLD and healthy control groups. The pooled results showed a significant decrease in alpha diversity in post-progressive period of NAFLD compared to the healthy control groups.

**Figure 5:**
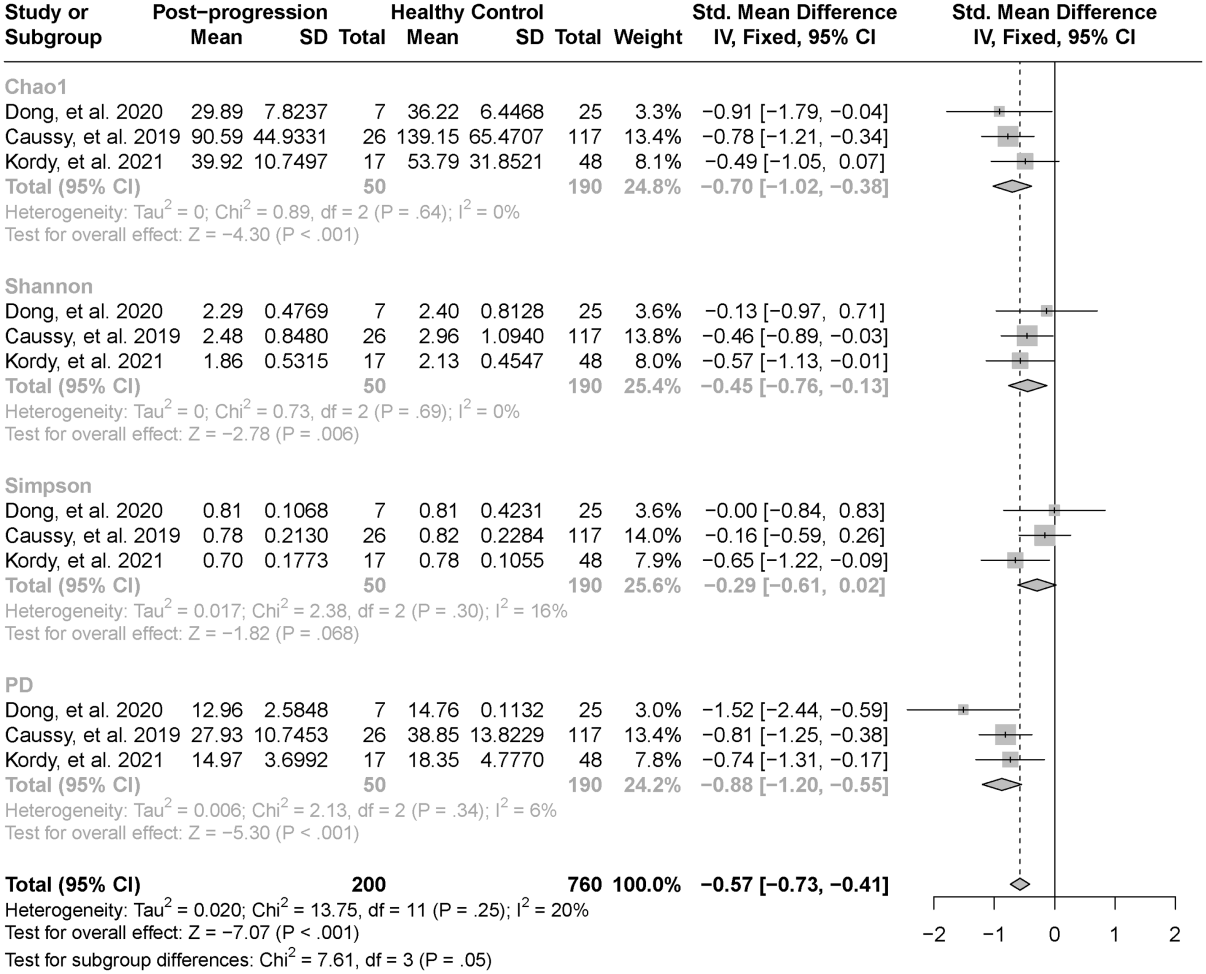
The forest plot shows the outcomes of a meta-analysis performed on the alpha-diversity of each parameter in both the pre-NAFLD and healthy control groups. The pooled results showed a significant decrease in alpha diversity in pre-progressive period of NAFLD compared to the healthy control groups.

As estimated by the Chao1, Shannon, and PD indexes, the gut microbial alpha diversity was reduced significantly in patients with NAFLD compared with healthy controls (all *p* < 0.01; Figure 2). The pooled estimate of all alpha diversity indices showed a significant decrease among patients with NAFLD (SMD = −0.32; 95% CI −0.42 to −0.21; *p* < 0.001; Figure 2). Furthermore, the pooled estimate showed a significant decrease in alpha diversity among patients with NAFLD compared with patients with obesity (*p* < 0.001; Supplementary Figure S1). As NAFLD progressed, there was a significant down regulation of alpha diversity (SMD = −0.39; 95% CI, −0.53 to −0.24; *p* < 0.001; Figure 3). When compared with healthy controls, the pooled estimates showed a significant decline in alpha diversity regardless of whether NAFLD was in the pre-progressive period (SMD = −0.27; 95% CI −0.39 to−0.16; *p* < 0.001; Figure 4) or the post-progressive period (SMD = −0.57; 95% CI −0.73 to−0.41; *p* < 0.001; Figure 5).

**Figure 6:**
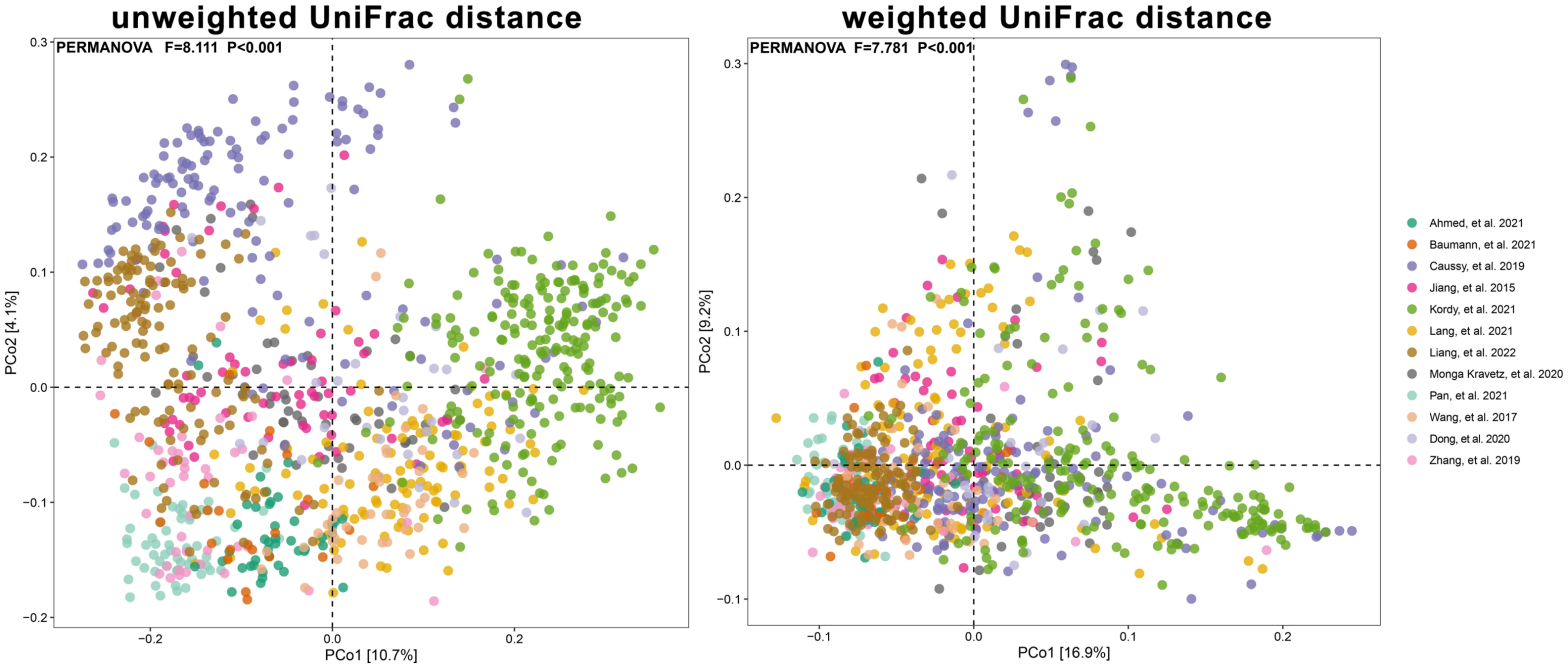
The PCoA plots showing unweighted UniFrac and weighted UniFrac distance as samples colored by study. Results of PERMANOVA test for significance between studies are shown on each plot.

**Figure 7:**
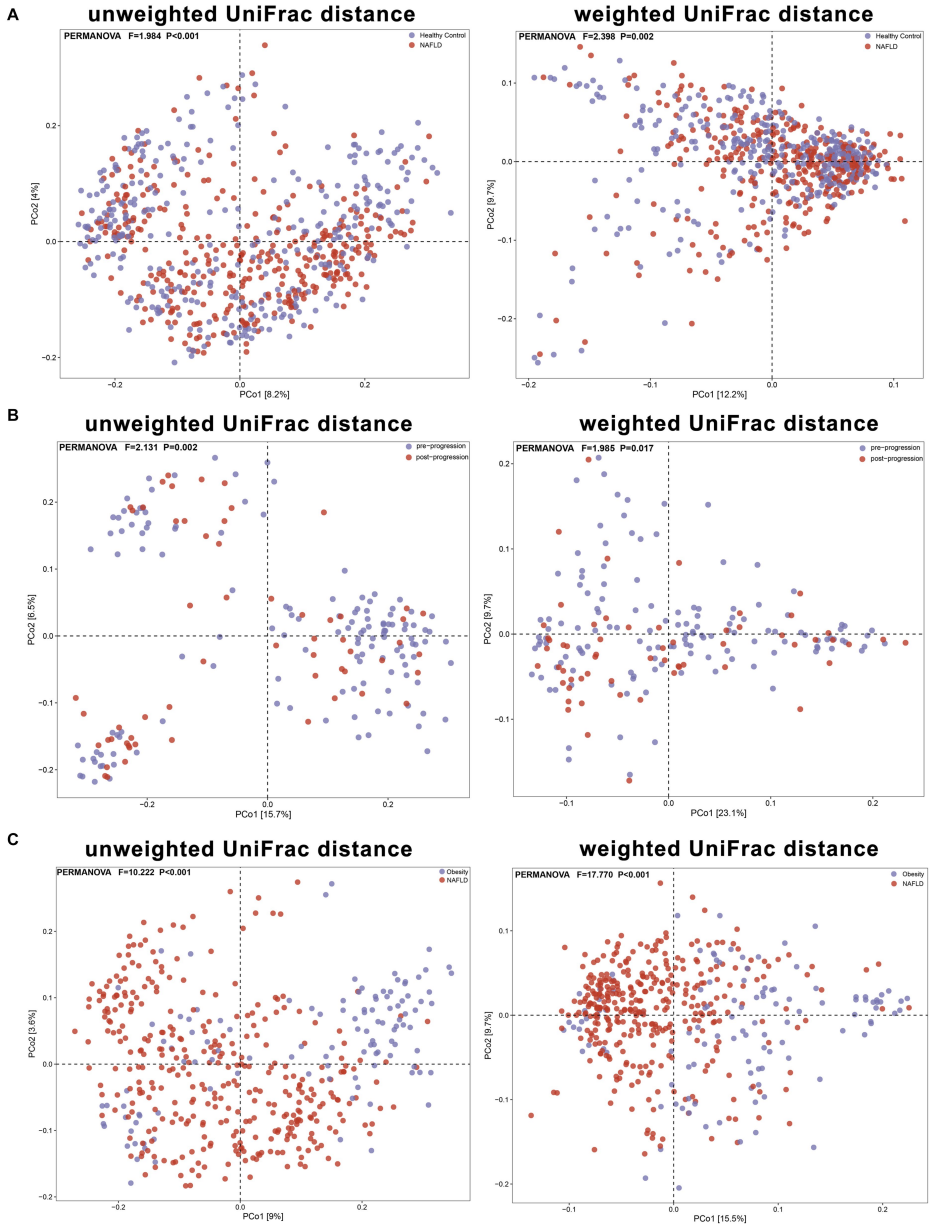
The PCoA plots showing unweighted UniFrac and weighted UniFrac distance as samples colored by group. Results of PERMANOVA test for significance between groups are shown on each plot. **(A)** Based on unweighted and weighted UniFrac distances, the PERMANOVA results reveal significant differences in beta diversity between the NAFLD group and the healthy control group (pseudo-F = 1.984, 2.398; *P* < 0.001, *P* = 0.002). **(B)** Based on unweighted and weighted UniFrac distances, the PERMANOVA results demonstrate significant differences in beta diversity between the pre-progressive and post-progressive stages of NAFLD (pseudo-F = 2.131, 1.985; *P* = 0.002, *P* = 0.017). **(C)** Based on unweighted and weighted UniFrac distances, the PERMANOVA results reveal significant differences in beta diversity between the obesity group and the NAFLD group (pseudo-F = 10.222, 17.770; *P* < 0.001, *P* < 0.001).

We aggregated the beta diversity analyses of the included studies according to the unweighted and weighted UniFrac distances. There were significant differences between samples when they were grouped by study or cases of NAFLD (PERMANOVA *p* < 0.001; Figures 6, 7). Furthermore, the PERMANOVA results based on unweighted and weighted UniFrac distances showed that the pseudo-*F* values in the different study groups (pseudo-*F* = 8.111, 7.781; *p* < 0.001, *p* < 0.001) were higher than in the different groups (Healthy Control and NAFLD pseudo-*F* = 1.984, 2.398; *p* < 0.001, *p* = 0.002; pre-progressive and post-progressive NAFLD pseudo-*F* = 2.131, 1.985; *p* = 0.002, *p* = 0.017). The interstudy variation outweighed the effect of NAFLD. We tested the effect of NAFLD for each study separately on the community composition by employing PCoA of multiple distance metrics and PERMANOVA for statistical testing. Except for the study by Zhang Y. et al. (2019), there was no significant influence of NAFLD on microbial community composition across all distance metrics in the remaining studies (all *p* > 0.05). However, in all other studies, NAFLD exhibited a significant effect on microbial composition in specific distance metrics (*p* < 0.05, PERMANOVA; Table 2; Supplementary Figure S2). In addition, we conducted PCoA based on the Bray–Curtis distance to evaluate the similarities in the gut microbiome composition among the groups in all included studies. Based on PERMANOVA, the results reported by Caussy et al. (2019), Zhang Y. et al. (2019), Dong et al. (2020), Kordy et al. (2021), and Lang et al. (2021) showed that the gut microbiome composition differed significantly between patients with NAFLD and healthy controls (*p* < 0.05). Of note, there were also significant differences in the gut microbial composition between pre-progressive and post-progressive NAFLD according to the results of Caussy et al. (2019), Dong et al. (2020), and Kordy et al. (2021) (*p* < 0.05).

**Table 2:** The beta diversity results of the included studies.

Study	Bray-Curtis		Jaccard		Unweighted uniFrac		Weighted uniFrac	
	R^2^	*p*	R^2^	*p*	R^2^	*p*	R^2^	*p*
Monga Kravetz et al., 2020	0.016	0.200	0.015	0.183	0.016	0.153	0.028	0.034^**^
Pan et al., 2021	0.027	0.117	0.027	0.119	0.028	0.325	0.039	0.029^*^
Jiang et al., 2015	0.015	0.093	0.014	0.025^*^	0.025	0.006^**^	0.015	0.221
Dong et al., 2020	0.096	<0.001^***^	0.073	<0.001^***^	0.075	0.01^**^	0.076	0.035^*^
Lang et al., 2021	0.012	0.045^*^	0.009	0.076	0.008	0.564	0.007	0.517
Caussy et al., 2019	0.027	<0.001^***^	0.019	<0.001^*^	0.027	<0.001^***^	0.039	0.002^***^
Zhang Y. et al. (2019)	0.033	0.071	0.030	0.190	0.027	0.721	0.034	0.274
Ahmed et al., 2021	0.018	1.000	0.018	1.000	0.022	0.015^*^	0.032	0.035^*^
Baumann et al., 2021	0.036	1.000	0.036	1.000	0.057	0.011^*^	0.069	0.017*
Wang et al., 2017	0.019	0.598	0.019	0.705	0.035	0.007^**^	0.033	0.074
Liang et al., 2022	0.008	0.464	0.008	0.403	0.008	0.425	0.016	0.027^*^
Kordy et al., 2021	0.026	<0.001^***^	0.019	<0.001^***^	0.025	<0.001^***^	0.058	<0.001^***^

^*^p < 0.05, ^**^p < 0.01, ^***^p < 0.001.

The Venn diagrams showing ASV intersections between groups indicated that there were differences in the gut microbial composition between groups based on the ASV levels (Supplementary Figure S3).

### 3.3 Phylogenetic profiles of the gut microbiome related to NAFLD

We analyzed the phylogenetic profiles of intestinal microorganisms between the NAFLD and healthy control groups. The average compositions and the top 10 highest relative abundances of the bacterial community in all groups at the phylum and genus levels are shown in Supplementary Figures S4, S5. The results from the studies of by Caussy et al. (2019), Dong et al. (2020), and Lang et al. (2021) showed a trend toward an increased abundance of *Firmicutes* and a decreased abundance of *Bacteroidetes* in patients with NAFLD; however, the results from the studies by Zhang Y. et al. (2019), and Kordy et al. (2021) showed a declining trend in *Firmicutes* in patients with NAFLD, while *Bacteroidetes* showed an increasing trend (Supplementary Figure S4). According to the results reported by Caussy et al. (2019), Zhang Y. et al. (2019), Dong et al. (2020), Kordy et al. (2021), and Lang et al. (2021), 18 genera including *Lactobacillus*, *Desulfovibrio*, *Escherichia-Shigella*, and *Veillonella* were significantly enriched, whereas 24 genera including *Christensenellaceae_R-7_group*, *Lachnospiraceae_UCG-001*, *Lachnospira*, and *Ruminococcaceae_UCG-002* were significantly reduced in patients with NAFLD compared with healthy controls (all *p* < 0.05; Supplementary Figure S6).

Furthermore, we compared the phylogenetic profiles of the gut microbiota between NAFLD and NAFLD-related fibrosis or liver cirrhosis at the class (Supplementary Figure S7), family (Supplementary Figure S8), and genus (Supplementary Figure S9) levels. At the class level, six bacterial populations including *Actinobacteria*, *Erysipelotrichia*, and *Methanobacteria*, were significantly enriched, whereas only *Bacilli* was significantly reduced in patients with NAFLD compared with patients with fibrosis or liver cirrhosis (all *p* < 0.05; Supplementary Figure S7). At the family level, nine bacterial populations including *Campylobacteraceae*, *Enterobacteriaceae*, and *Prevotellaceae* were significantly enriched, whereas two bacterial populations, namely *Bacteroidaceae* and *Peptostreptococcaceae*, were significantly reduced in patients with NAFLD (all *p* < 0.05; Supplementary Figure S8). At the genus level, 18 bacterial populations including *Alloprevotella*, *Escherichia-Shigella*, and *Prevotella_2* were significantly increased, whereas seven bacterial populations including *Colidextribacter*, *Bacteroides*, and *Faecalibacterium* were significantly decreased in the pre-progressive period of NAFLD compared with the post-progressive period of NAFLD (all *p* < 0.05; Supplementary Figure S9).

### 3.4 Crucial microbiota and microbial functions related to NAFLD

**Figure 8:**
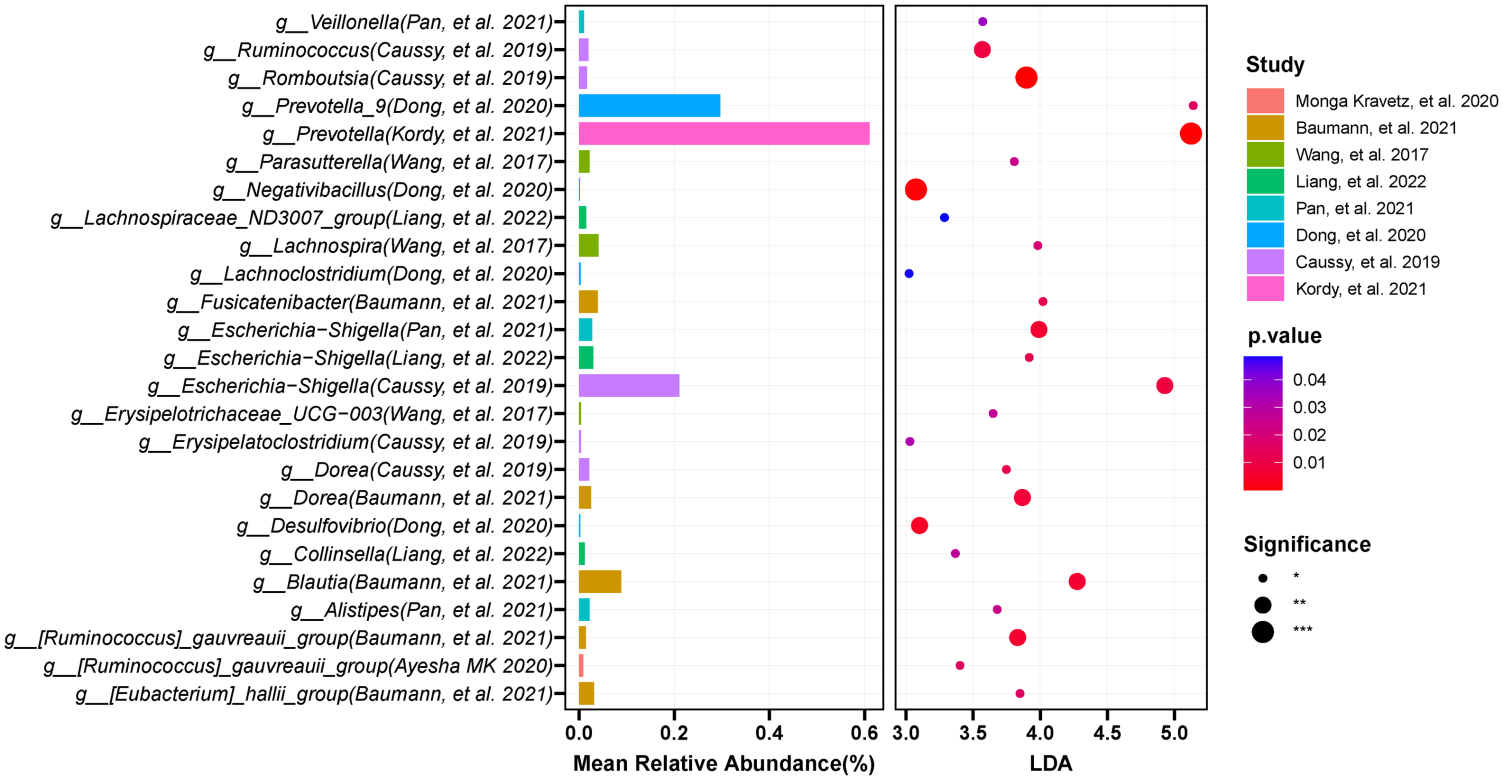
The integration of LEFSE-based and random forestation methods identifies crucial genera that significantly increase in the occurrence or development of NAFLD. Based on the linear discriminant analysis (LDA score ≥ 3 and *p* < 0.05) and Random Forests selection, 22 genera including Desulfovibrio, *Prevotella_9*, *Dorea*, *Escherichia-Shigella*, and *Negativibacillus* were significantly enriched in NAFLD.

**Figure 9:**
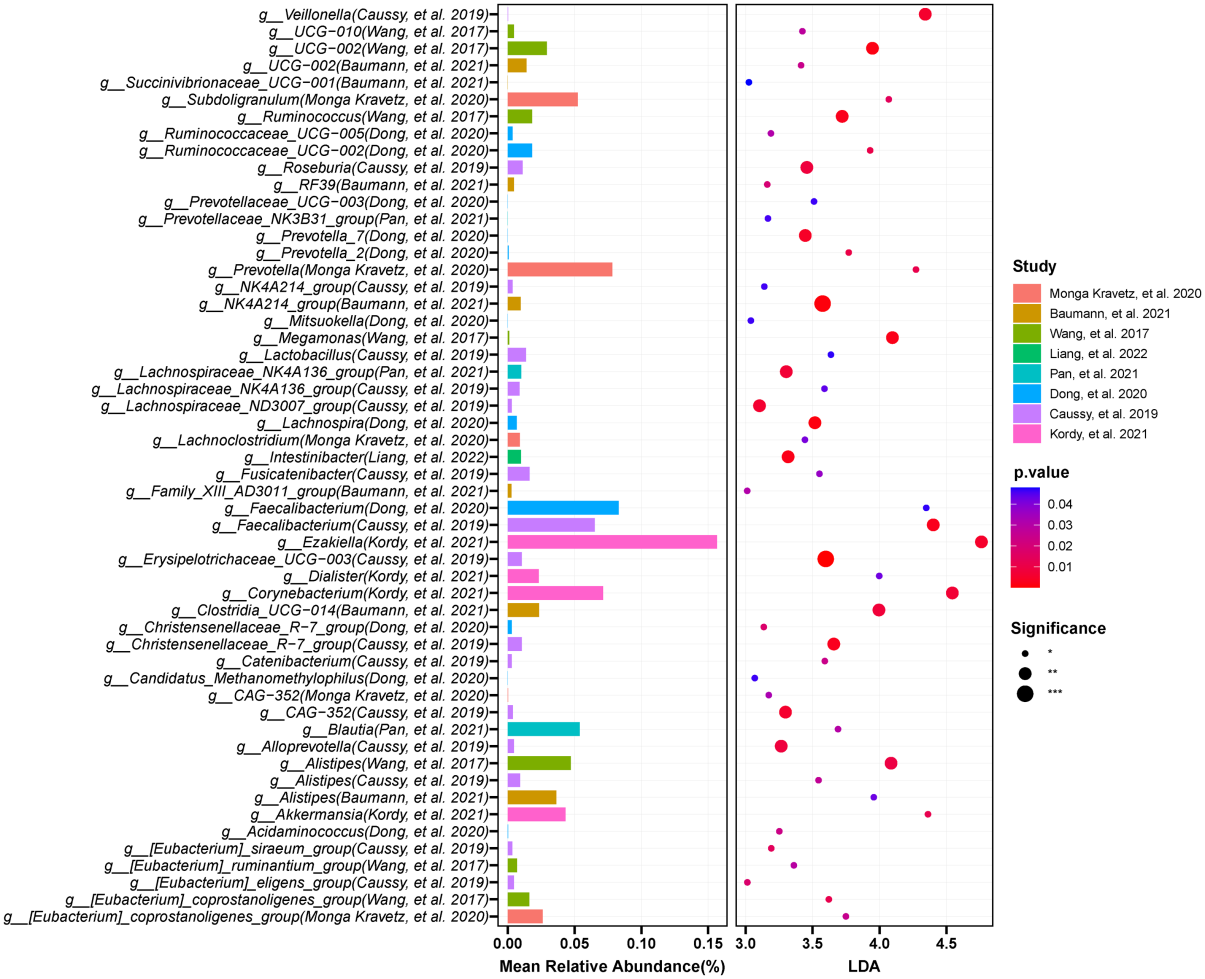
The integration of LEFSE-based and Random Forest methods identifies crucial genera that significantly decreased in the occurrence or development of NAFLD. Through LDA score ≥ 3 and *p* < 0.05, in combination with Random Forests selection, and by requiring at least three studies to appear the findings, the results were obtained: 46 genera including *Faecalibacterium*, *Lachnospira*, *Catenibacterium*, and *UCG-002* were significantly reduced in NAFLD compared with those in the controls.

We used LEfSe to determine the maximum difference in the microbial structures in patients with NAFLD, versus those in healthy controls or patients with obesity. Then random forest methods to validate the LEfSe results. We screened the 12 included studies separately for dominant biomarkers by employing LEfSe. Based on an LDA score ≥ 3 and *p* < 0.05 and random forest selection, we found 22 genera including *Desulfovibrio*, *Prevotella_9*, *Dorea*, *Escherichia-Shigella*, and *Negativibacillus* that were significantly enriched (*p* < 0.05; Figure 8), while 46 genera including *Faecalibacterium*, *Lachnospira*, *Catenibacterium*, *Lactobacillus*, and *UCG-002* were significantly reduced in patients with NAFLD (*p* < 0.05; Figure 9), compared with healthy controls or patients with obesity. More details of the LEfSe and random forest analyses are shown in Supplementary Figures S10, S11.

**Figure 10:**
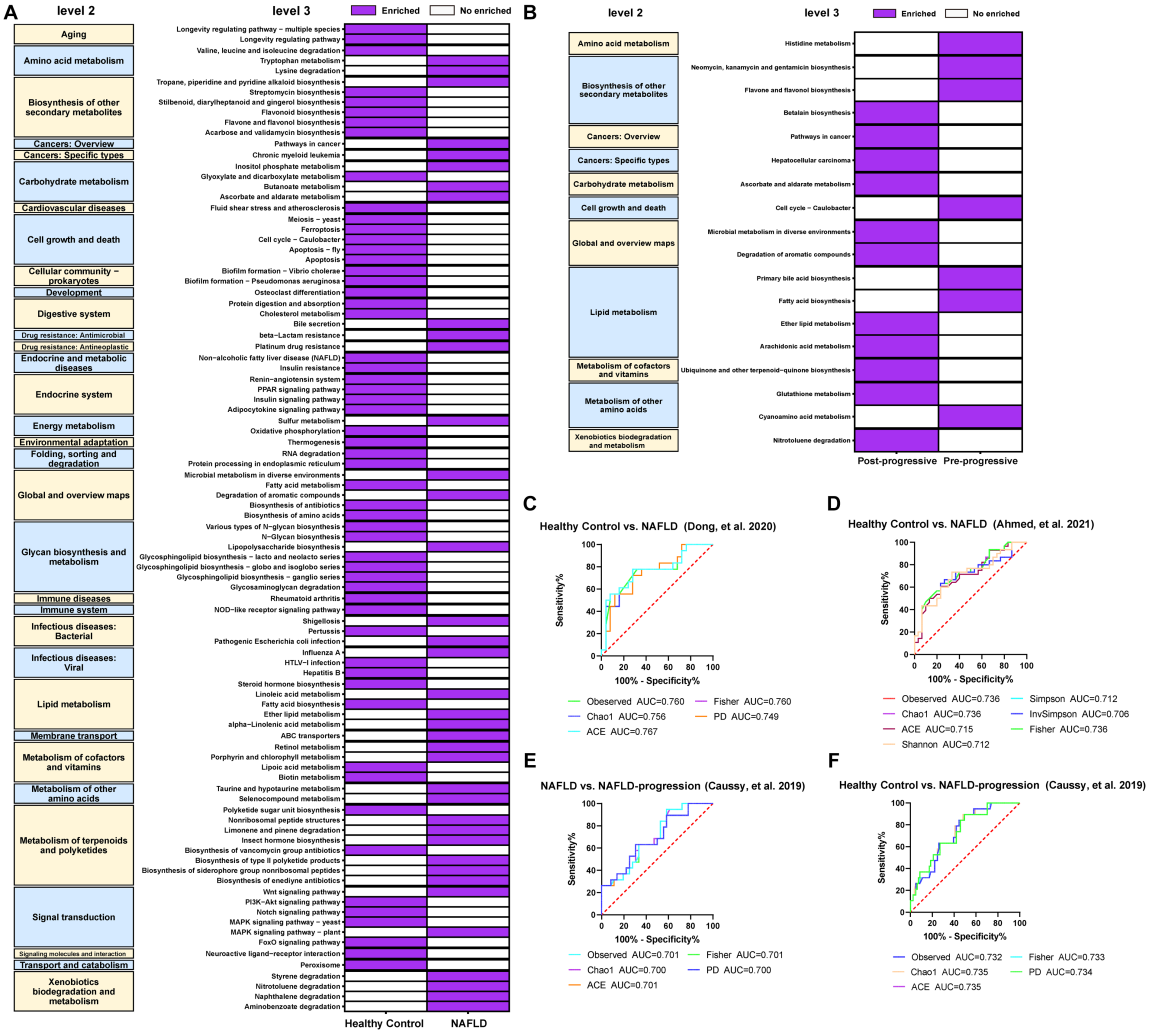
Crucial microbial functional pathways associated with the **(A)** occurrence and **(B)** progression of NAFLD and required to be present in at least two studies. The results showed that 56 predicted microbial functions including lipopolysaccharide biosynthesis, tryptophan metabolism, ether lipid metabolism, alpha-Linolenic acid metabolism, and linoleic acid metabolism were remarkably increased compared with those in the healthy controls. As the NAFLD progresses, we observed a significant increase in 11 predicted microbial functions, such as arachidonic acid metabolism, ether lipid metabolism, and glutathione metabolism, compared to post-NAFLD. **(C)** Five diversity indexes reached AUC values over 0.7 between NAFLD (no fibrosis) patients (*n* = 16) versus healthy controls (*n* = 25) based on the dataset of PRJNA542724 (Dong et al., 2020). **(D)** Seven diversity indexes reached AUC values over 0.7 between NAFLD patients (*n* = 29) versus healthy controls (*n* = 30) based on the dataset of PRJNA682382 (Ahmed et al., 2021). **(E)** Five diversity indexes reached AUC values over 0.7 between NAFLD patients (*n* = 51) versus healthy controls (*n* = 117) based on the dataset of PRJEB28350 (Caussy et al., 2019). **(F)** Five diversity indexes reached AUC values over 0.7 between NAFLD-CIR patients (*n* = 26) versus healthy controls (*n* = 117) based on the dataset of PRJEB28350 (Caussy et al., 2019).

We constructed the KEGG pathway profiles by using Tax4fun2 version 1.1.5 and used the 16S rRNA gene sequences to predict the microbial community function. The gut microbial community function profiles and the predominant microbial functions in patients with NAFLD and healthy controls are shown with a heatmap (Figure 10A). Thirty-eight predicted microbial functions including lipopolysaccharide (LPS) biosynthesis, tryptophan metabolism, ether lipid metabolism, alpha-linolenic acid metabolism, and linoleic acid metabolism were remarkably increased, while 56 predicted microbial functions including linoleic acid metabolism, glyoxylate, and dicarboxylate metabolism, biosynthesis of amino acids, fatty acid metabolism, and cholesterol metabolism were remarkably decreased in patients with NAFLD compared with healthy controls (*p* < 0.05; Figure 10A). Subsequently, we analyzed the predicted differential functional KEGG pathways related to NAFLD progression. As NAFLD progresses, 11 predicted microbial functions including arachidonic acid metabolism, ether lipid metabolism, and glutathione metabolism were remarkably increased, while seven predicted microbial functions including histidine metabolism, primary bile acid biosynthesis, cyanoamino acid metabolism, and fatty acid biosynthesis were remarkably decreased in the pre-progressive period of NAFLD, compared with the post-progressive period of NAFLD (*p* < 0.05; Figure 10B).

### 3.5 Alpha diversity and gut microbial markers may indicate a higher risk of NAFLD occurrence or progression.

We conducted ROC curve analysis to evaluate the potential of the gut microbiota as a non-invasive predictive indicator to assess the risk of occurrence and progression of NAFLD. The predictive risk capacity is considered accurate for an AUC > 0.7 and *p* < 0.05. In the model predicting NAFLD occurrence and NALFD progression, all alpha diversity metrics reached an AUC > 0.7 (*p* < 0.05; Figures 10C–F). The observed, Chao1, and ACE indexes reached an AUC of 0.760, 0.756, and 0.767, respectively, in the model predicting the occurrence of NAFLD (Figures 10C,D). The observed, Chao1, and ACE indexes reached an AUC of 0.701, 0.700, and 0.701, respectively, in the model predicting the progression of NAFLD (Figure 10E). Besides, the observed, Chao1, and ACE indexes reached an AUC of 0.732, 0.735, and 0.735, respectively, in the model comparing healthy controls and the NALFD progression groups (Figure 10F).

**Figure 11:**
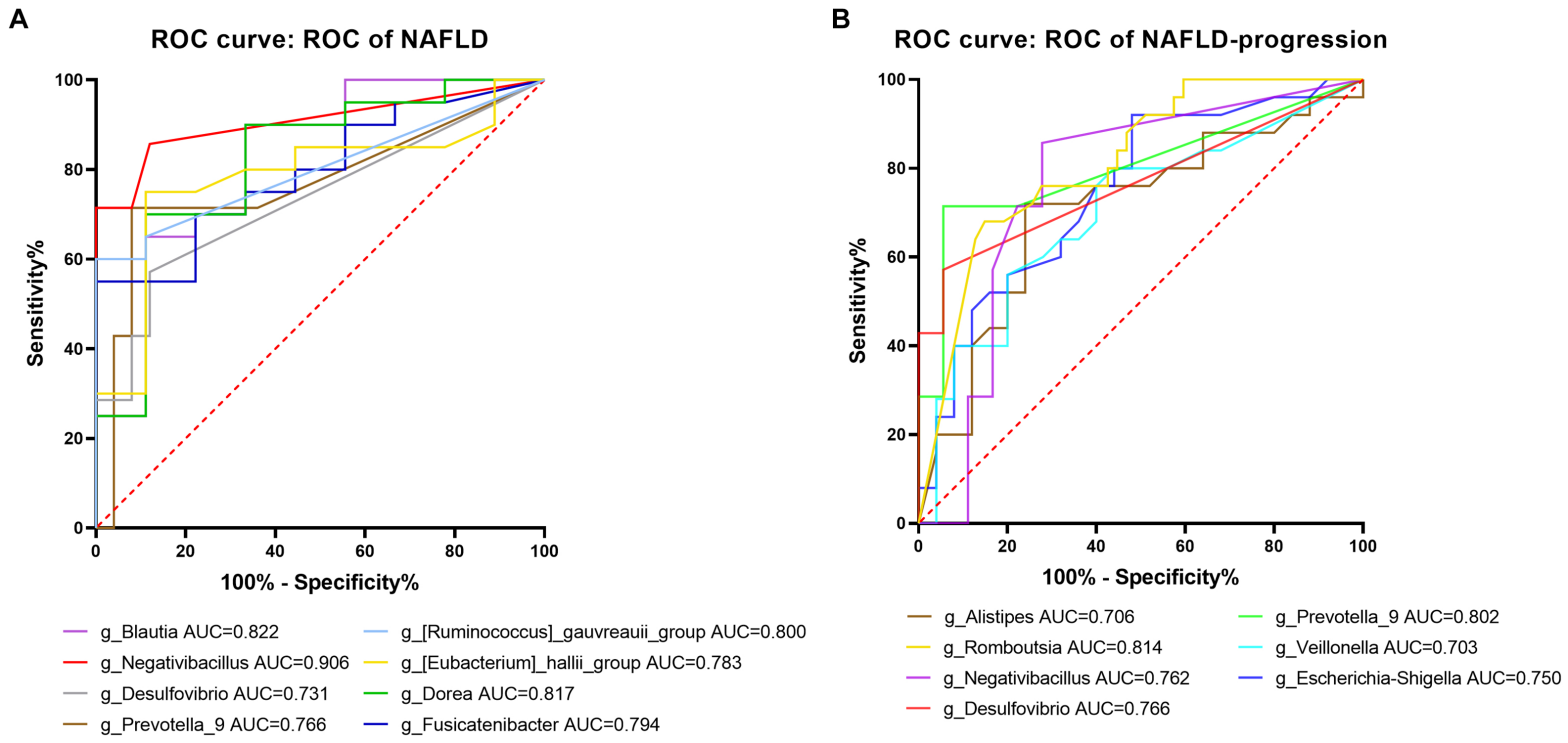
Predictive potential of bacteria at the genus level in NAFLD occurrence and NAFLD progress. **(A)** ROC curve for NAFLD compared to healthy controls. **(B)** ROC curve comparing the post-progressive period of NAFLD to the pre-progressive period of NAFLD. AUC (0.5–0.7), low accuracy; AUC (0.7–0.9), moderate accuracy; AUC (> 0.9), high accuracy.

At the genus level, eight significantly different gut microbes, including *Negativibacillus*, *Prevotella_9*, *Blautia*, and *Desulfovibrio*, based on LefSe, had a highly accurate predictive risk capacity to predict NAFLD occurrence (AUC > 0.7, *p* < 0.05, Figure 11A). *Negativibacillus*, *Blautia*, *Dorea,* and *gauvreauii_group* had an AUC of 0.907, 0.822, 0.817, and 0.800, respectively, in the model predicting NAFLD occurrence. Seven genera including *Negativibacillus*, *Prevotella_9*, *Desulfovibrio,* and *Veillonella* had a highly accurate predictive risk capacity to predict NAFLD progression (Figure 11B). Among them, *Romboutsia*, *Prevotella_9*, *Desulfovibrio*, and *Negativibacillus* had an AUC of 0.814, 0.802, 0.766, and 0.762, respectively, in the model predicting NAFLD progression. It is worth mentioning that the AUC was >0.7 for *Negativibacillus*, *Prevotella_9*, and *Desulfovibrio* in the models predicting NAFLD occurrence and NAFLD progression.

## 4 Discussion

NAFLD is a growing global phenomenon and is considered to be a complex disease associated with the dysregulation of multiple interconnected biological pathways (Li et al., 2021). In recent years, there has been increased research attention regarding the relationship between gut dysbiosis and the occurrence and progression of NAFLD. Many studies have been conducted to demonstrate gut microbiota dysregulation in patients with NAFLD compared with healthy controls (Boursier et al., 2016; Zhou et al., 2017; Nishiyama et al., 2020; Long et al., 2021; Shi et al., 2021). However, previous studies have focused on Europe and the Americas (particularly North America), and there are few studies from Asia. Regional differences in diet and culture impact the composition and alterations of the gut microbiota (Dai et al., 2020), resulting in inconsistent findings. There is a lack of consensus among the existing studies on gut microbiota changes in patients with NAFLD. Our study provides a comprehensive assessment of the gut microbiota composition in the context of the occurrence and progression of NAFLD. We aimed to identify specific gut microbiota biomarkers and to clarify the intricate relationship between the gut microbiota and NAFLD pathogenesis.

In our overall evaluation of alpha diversity across the 10 datasets, we found a significant reduction in intestinal alpha microbial diversity in patients with NAFLD compared with healthy controls. This finding indicates that alterations in the abundance and diversity of the gut microbiome are strongly related to NAFLD, consistent with the previous research results (Mouzaki et al., 2013; Zhu et al., 2013; Kim et al., 2019). When restricting the control groups to patients with obesity, there were still significant reductions in alpha diversity among patients with NAFLD compared with controls. This phenomenon indicates that although obesity may affect the diversity of the gut microbiome (Sarmiento et al., 2022), NALFD could play a more crucial role in decreasing the gut microbial diversity.

The Shannon index, an indicator of the diversity of bacterial populations, declined significantly after the progression of NAFLD, indicating that intestinal diversity also decreased as NAFLD progressed. Alpha diversity serves as a crucial indicator to evaluate the health of the intestinal microbiota. It reflects the microbiome richness and community diversity of the intestine. A decrease in microbial alpha diversity can potentially contribute to the pathogenesis and progression of diseases (Wenhui et al., 2022).

Our ROC analysis using alpha diversity as a predictor yielded a high AUC scores were observed for the pre-progressive and post-progressive periods of NAFLD. These findings suggest that alpha diversity exhibits a promising predictive ability and may serve as a potential risk predictor for NAFLD. Our analysis of beta diversity revealed significant technical and experimental variations among the studies. Differences in sequencing methods can impact taxonomic resolution and potentially alter compositional features. These “study effects” have a substantial impact on microbial community composition, but estimating individual effects is challenging due to the potential issue of multicollinearity among variables.

Subsequently, we conducted PCoA using Bray–Curtis distances to examine beta diversity and observed significant variations only in some datasets. Notably, there were significant changes in the composition of the gut microbiota as NAFLD progressed, while there were no significant differences during the pre-progressive period of NAFLD. These findings are consistent with those reported by Dong et al. (2020) and Caussy et al. (2019); these samples were obtained from Europe and the United States. Although samples from Asia also included patients with post-progressive NAFLD, we did not observe significant differences in our study.

At the phylum level, the human gut microbiota is mainly composed of *Bacteroidetes* and *Firmicutes* (Mo et al., 2022). In the European and American cohort studies, we observed a trend toward an increased abundance of *Firmicutes* and a decreased abundance of *Bacteroidetes* in patients with NAFLD, but the differences did not reach statistical significance. In contrast, the Asian studies showed a significant decrease in *Firmicutes* and a significant increase in *Bacteroidetes* in patients with NAFLD. This result is consistent with the study by Tsai et al. (2020), The authors observed that individuals with a body mass index (BMI) greater than 25 kg/m^2^ had significantly higher proportions of *Bacteroidetes* after controlling for BMI factors (Tsai et al., 2020). Notably, non-obese individuals with NAFLD exhibited a reduction in the *Firmicutes-*to-*Bacteroidetes* ratio (Wang et al., 2019). This observation challenges the conventional view that a high *Firmicutes*-to-*Bacteroidetes* ratio is a crucial characteristic of NAFLD (Xing et al., 2019). The Western diet is characterized by high levels of fat, protein, and sugar, while the Asian diet is typically low in fat and protein, but rich in dietary fiber. Similarly, the Latino diet mainly consists of vegetables, nuts, grains, and low-fat red or processed meat (Wang et al., 2021). This difference in diet composition has a significant impact on the growth of the phylum *Bacteroidetes*; these organisms are associated with the production of branched-chain fatty acids through amino acid fermentation, which can lead to insulin resistance and promote the development of NAFLD (Newgard, 2012; Tsai et al., 2020). Expanding on this comparison revealed varying trends in *Bacteroidetes* and *Firmicutes* abundance in patients with NAFLD across geographical regions, highlighting the potential influence of dietary habits on the gut microbiota composition. Interestingly, we observed an enrichment of lactic acid-producing bacteria (such as *Lactobacillus*) in patients with NAFLD, consistent with the microbiome results reported by Min et al. (2022) in patients with bladder cancer. Although there is an association between NAFLD and bladder cancer (Bjorkstrom et al., 2022), the complex mechanisms through which NAFLD promotes the occurrence of bladder cancer remain to be fully elucidated. Given the pivotal role of the microbiome in the development and progression of cancer (Wu et al., 2018), the relationship between NAFLD and bladder cancer might be clarified by further analysis of the microbiome. Subsequently, we identified 22 dominant genera in NAFLD using LEfSe and random forest analyses. Among them, *Escherichia-Shigella* could evade host immune surveillance and induce inflammation in the intestine by inhibiting autophagy in epithelial and inflammatory cells (Liang et al., 2022). *Desulfovibrio*, a bacterial genus purported to promote inflammation, synthesizes hydrogen sulfide that exhibits cytotoxicity toward intestinal cells (Zheng et al., 2018; He et al., 2020). Notably, *Desulfovibrio* is associated with LPS biosynthesis, and an increase in LPS levels triggers a systemic inflammatory response. Elevated levels of LPS are crucial for both the occurrence and progression of NAFLD (Diling et al., 2017). *Prevotella* can ferment carbohydrates, resulting in the production of short-chain fatty acids that reduce hepatic lipogenesis, potentially providing relief from the development of NAFLD (Gaike et al., 2020; Luo et al., 2021). This implies that gut microbiome disturbances may trigger compensatory mechanisms both before and after NAFLD progression. *Prevotella*, as a common probiotic, plays a crucial role in regulating gut health (Liu et al., 2019). Focusing on *Prevotella* could become a promising direction for NAFLD treatments, perhaps through the development of specific probiotic formulations, the implementation of targeted dietary interventions, or the exploration of alternative approaches to modulate gut microbiota balance. However, caution is warranted because this cannot be regarded as conclusive evidence of compensatory mechanisms. Additional validation experiments are necessary to elucidate the compensatory mechanisms of *Prevotella* in the development and progression of NAFLD. Moreover, ROC analysis identified 13 genera, including *Desulfovibrio*, *Prevotella*, *Negativibacillus*, and *Escherichia-Shigella*, with preferable diagnostic potential to distinguish patients with NAFLD from healthy controls and to predict NAFLD progression. The diagnostic power of a single genus in predicting the occurrence and progression of NAFLD is limited (Fenn et al., 2022). Therefore, machine learning could be utilized to build a potential risk prediction model based on the combination of many critical genera or metabolites for assessing the risk of NAFLD.

Based on the predicted function analysis, LPS, tryptophan metabolism, and various fatty acid metabolism pathways were significantly enriched in patients with NAFLD. Additionally, glutathione metabolism was enriched in patients with post-progressive NAFLD. Interestingly, in a cohort study examining NAFLD in adolescents, glutathione metabolism declined significantly with the development of NAFLD (Xanthakos et al., 2015), suggesting that the dysregulation of glutathione metabolism is closely associated with the pathological conditions of NAFLD. However, the majority of studies have focused on glutathione synthesis rather than glutathione redox balance (Gansemer et al., 2020). Glutathione, the primary antioxidant molecule involved in oxidative defense mechanisms (Kang et al., 2018), exists in the body primarily in reduced glutathione (GSH) and oxidized glutathione (GSSG) forms. GSH can be converted to GSSG by glutathione reductase. The progression of NAFLD is significantly influenced by oxidative stress caused by an imbalance between the production of reactive oxygen species (ROS) and antioxidant defenses (Hong et al., 2021). Due to the accumulation of hepatic lipids in patients with NAFLD, liver ROS levels are elevated (Oh and Chun, 2022). Excess ROS disrupts lipid metabolism and inhibits the activity of antioxidant enzymes (Hong et al., 2021), leading to the accumulation of intracellular GSSG. Elevated levels of GSSG promote the progression of NAFLD to an inflammatory state by inducing the formation of glutathionylated IKK-β (IKK-β-SSG), which inhibits nuclear factor κB and increases the expression of the pro-inflammatory cytokine tumor necrosis factor α (Dou et al., 2018). Therefore, we postulate that alterations in these crucial gut microbes impact the production of diverse metabolites within the body, including LPS and glutathione metabolism, thereby promoting the onset and progression of NAFLD.

We conducted a comprehensive investigation of studies from diverse regions, including healthy controls, patients with obesity, and, and patients with biopsy-confirmed NAFLD, to provide more meaningful findings. However, this study has a few limitations that need to be acknowledged. First, due to insufficient information on detailed sample characteristics such as age, BMI, and diet, we could not conduct further stratified analysis. Additionally, it should be noted that each study used different diagnostic methods and criteria for NAFLD, leading to some heterogeneity among the studies. Moreover, we could not include some articles in the integrated analysis due to the unavailability of the original data. This constitutes a major limitation of our study because we could discuss the findings from these articles but not include them in our comprehensive analysis. Second, the decision to use 16S rRNA gene sequencing data instead of macro-genomic sequencing data limited the accuracy of species-level and functional analyses. Additionally, differences in the choice of sequencing instruments and variable regions sequenced across studies may have introduced confounding factors that could have biased the results. we urge caution regarding the interpretation of our findings. Finally, this study is cross-sectional and only explored the correlation between the gut microbiota and NAFLD. We cannot draw conclusions about causality, which will require the use of *in vitro* experiments to illustrate the cause-effect relationship between crucial bacteria and NAFLD.

## 5 Conclusion

In summary, our results demonstrate that gut microbial diversity declines significantly with the progression of NAFLD, while alpha diversity exhibits promise as a predictive indicator for NAFLD risk. The increased abundance of *Desulfovibrio*, *Negativibacillus*, *Prevotella*, *Escherichia-Shigella*, and other genera may serve as an indication of their predictive risk ability for NAFLD progression. With the occurrence and progression of NAFLD, changes in functional pathways such as LPS metabolism, glutathione metabolism, and lipid metabolism are significantly upregulated. These findings are important for elucidating the relationship between the gut microbiota and NAFLD, and are crucial for elucidating the pathogenesis, prevention, and treatment of this disease.

## Data availability statement

The original contributions presented in the study are included in the article/Supplementary material, further inquiries can be directed to the corresponding authors.

## Ethics statement

Ethical approval was not required for the study involving humans in accordance with the local legislation and institutional requirements. Written informed consent to participate in this study was not required from the participants or the participants' legal guardians/next of kin in accordance with the national legislation and the institutional requirements.

## Author contributions

HM: Writing – original draft. XY: Writing – original draft, YX: Data curation, Formal analysis, Visualization, Writing – review & editing. JZ: Visualization, Writing – review & editing. QW: Writing – review & editing. YW: Writing – review & editing. YY: Data curation, Writing – review & editing. DL: Data curation, Writing – review & editing. LY: Supervision, Writing – review & editing. PC: Supervision, Writing – review & editing. HL: Conceptualization, Writing – review & editing. JH: Conceptualization, Funding acquisition, Writing – review & editing.

## Footnotes

^1^https://pubmed.ncbi.nlm.nih.gov/

^2^https://www.ncbi.nlm.nih.gov/bioproject

^3^http://ncbi.github.io/sra-tools/

^4^https://qiime2.org

^5^https://github.com/qiime2/q2-cutadapt

^6^https://github.com/vegandevs/vegan

^7^https://CRAN.R-project.org/package=VennDiagram

^8^https://ggplot2.tidyverse.org/

^9^http://CRAN.R-project.org/package=meta
