# Supplementary material for: The role of gut microbiota in the occurrence and progression of non-alcoholic fatty liver disease
Source: Front Microbiol. 2024 Jan 5;14:1257903. doi: 10.3389/fmicb.2023.1257903 (PMC10797006; doi:10.3389/fmicb.2023.1257903)
Supplement: Supplementary file 1 [file Table_1.docx]

| **Table S1 Search Strategy** | | |
| --- | --- | --- |
| No. | Terms | Comments |
| #1 | "nonalcoholic fatty liver disease"[MeSH Terms] OR "nafld*"[All Fields] OR "nonalcoholic steatosis*"[All Fields] OR "nash*"[All Fields] OR "simple hepatic steatosis*"[All Fields] OR "nafl*"[All Fields] | non-alcoholic fatty liver disease |
| #2 | "gut"[Text Word] OR "intestinal*"[Text Word]) AND "microbiota"[MeSH Terms]) OR "microbe*"[Text Word] OR "microbiome*"[Text Word] OR "flora*"[Text Word] OR "microorganism*"[Text Word] OR "microflora*"[Text Word] | microbiota |
| #3 | (#1 AND #2) |  |
