# Supplementary material for: The role of gut microbiota in the occurrence and progression of non-alcoholic fatty liver disease
Source: Front Microbiol. 2024 Jan 5;14:1257903. doi: 10.3389/fmicb.2023.1257903 (PMC10797006; doi:10.3389/fmicb.2023.1257903)
Supplement: Supplementary file 2 [file Table_2.docx]

| **Table S2 Diagnostic methods and criteria for NAFLD in the included studies** | | | |
| --- | --- | --- | --- |
| **Author, Year** | **Diagnostic methods for NAFLD** | **Definition of control group** | **Definition of NAFLD group** |
| Monga Kravetz, et al. 2020 | MRI | Obesity: BMI>95th percentile | HFF≥5.5% |
| Pan, et al. 2021 | Ultrasound | NA | NA |
| Jiang, et al. 2015 | Imaging or Histology | Healthy Control | based on American Association for the Study of Liver Diseases Practice Guidelines |
| Dong, et al. 2020 | Ultrasound and FibroScan | Healthy Control | NAFLD no fibrosis: F0/F1^a^  NAFLD fibrosis: F2 to F4^a^ |
| Lang, et al. 2021 | Ultrasound | Healthy Control | HFF≥5.0% |
| Caussy, et al. 2019 | MRI | Healthy Control | NAFLD: MRI-PDFF≥5%^b^  NAFLD-cirrhosis: MRE threshold≥3.63 kPa^b^ |
| Zhang, et al. 2019 | Ultrasound | Healthy Control | Ultrasonography-proven |
| Ahmed, et al. 2021 | MRI-PDFF^c^ | Healthy Control | MRI-PDFF-proven |
| Baumann, et al. 2021 | Ultrasound | Healthy Control | Ultrasonography-proven |
| Wang, et al. 2017 | NA | Healthy Control | NA |
| Liang, et al. 2022 | Ultrasound | Healthy Control | Ultrasonography-proven |
| Kordy, et al. 2021 | Percutaneous liver biopsy | Obesity: BMI≥85th percentile | NAFL: ALT^d^≥50 IU/L males, ≥44 IU/L females  NASH: based on NAS^e^ |

a. Standard cutoffs of liver stiffness as measured in kilopascals was used to determine extent of liver fibrosis (F0/F1 to F4).

b. Based on American Association for the Study of Liver Study Practice Guidelines.

c. MRI-PDFF: MRI-derived proton density fat fraction.

d. alanine aminotransferase (ALT)

e. based on NAFLD activity score (NAS) on clinically-indicated percutaneous liver biopsy as proposed by Kleiner et al. (2005)
