# Supplementary material for: The role of gut microbiota in the occurrence and progression of non-alcoholic fatty liver disease
Source: Front Microbiol. 2024 Jan 5;14:1257903. doi: 10.3389/fmicb.2023.1257903 (PMC10797006; doi:10.3389/fmicb.2023.1257903)
Supplement: Supplementary file 3 [file Table_3.docx]

| **Table S3 The reads were trimmed using the following parameters in the DADA2 plugin for the each studies** | | | | |
| --- | --- | --- | --- | --- |
| **Study** | **--p-trim-left-f** | **--p-trunc-len-f** | **--p-trim-left-r** | **--p-trunc-len-r** |
| Monga Kravetz, et al. 2020 | 9 | 250 | 8 | 250 |
| Pan, et al. 2021 | 0 | 250 | 0 | 250 |
| Jiang, et al. 2015 | 38 | 130 | 30 | 130 |
| Dong, et al. 2020 | 0 | 250 | 0 | 241 |
| Lang, et al. 2021^*^ | --p-trim-left 0 | --p-trunc-len 297 |  |  |
| Caussy, et al. 2019^*^ | --p-trim-left 9 | --p-trunc-len 151 |  |  |
| Zhang, et al. 2019 | 0 | 250 | 0 | 250 |
| Ahmed, et al. 2021 | 13 | 250 | 13 | 216 |
| Baumann, et al. 2021 | 0 | 250 | 0 | 250 |
| Wang, et al. 2017 | 10 | 291 | 6 | 230 |
| Liang, et al. 2022 | 6 | 300 | 7 | 297 |
| Kordy, et al. 2021 | 6 | 150 | 8 | 149 |

Paired-end sequencing was used with the DADA2 "qiime dada2 denoise-paired" command in QIIME 2.

^*^ Single read sequencing was used with the DADA2 " qiime dada2 denoise-single" command in QIIME 2 with parameters "--p-trim-left" and "--p-trunc-len".
