# Supplementary figures and images for: The role of gut microbiota in the occurrence and progression of non-alcoholic fatty liver disease
Source: Front Microbiol. 2024 Jan 5;14:1257903. doi: 10.3389/fmicb.2023.1257903 (PMC10797006; doi:10.3389/fmicb.2023.1257903)

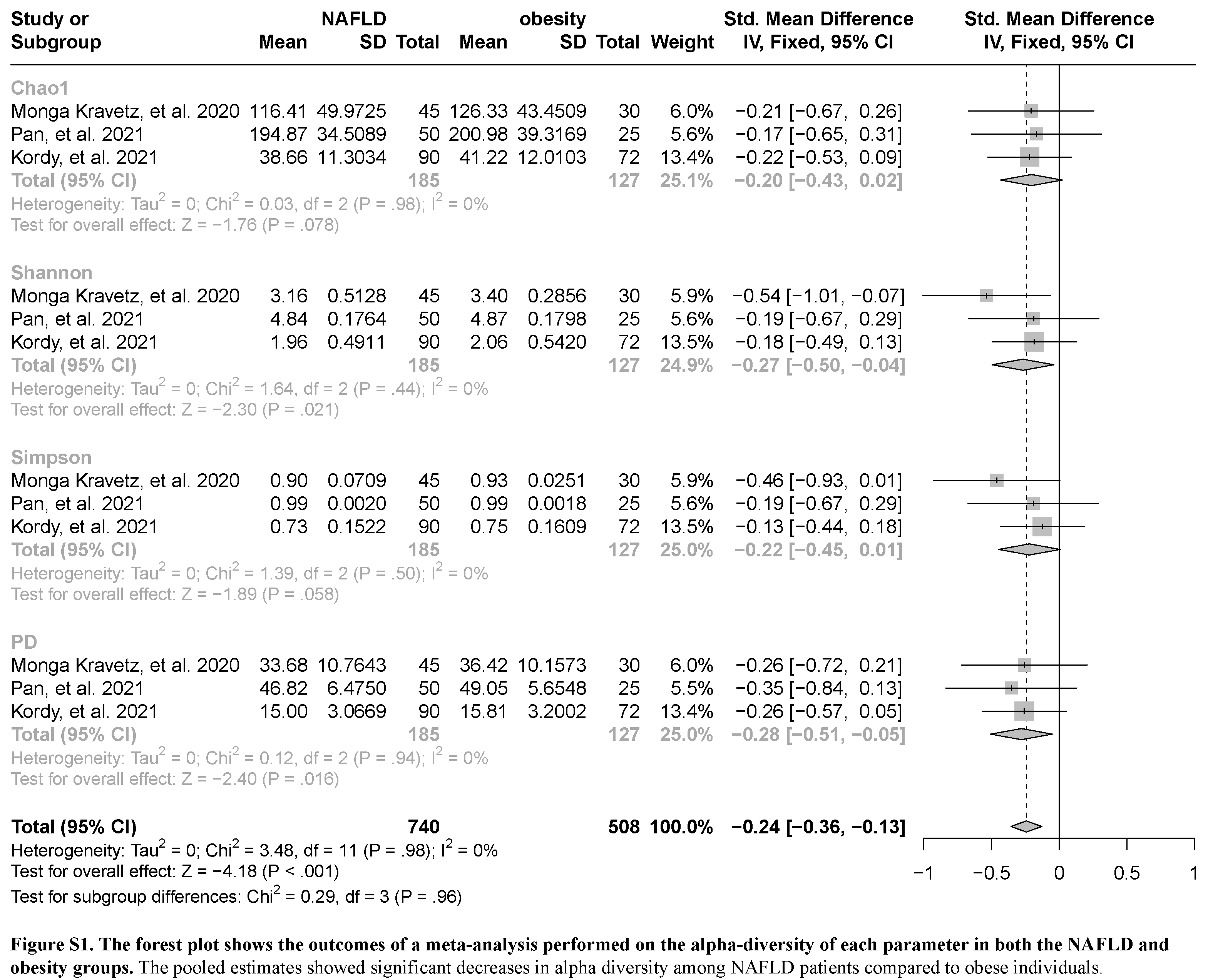

Supplement: Supplementary file 4 [file Image_1.tif]

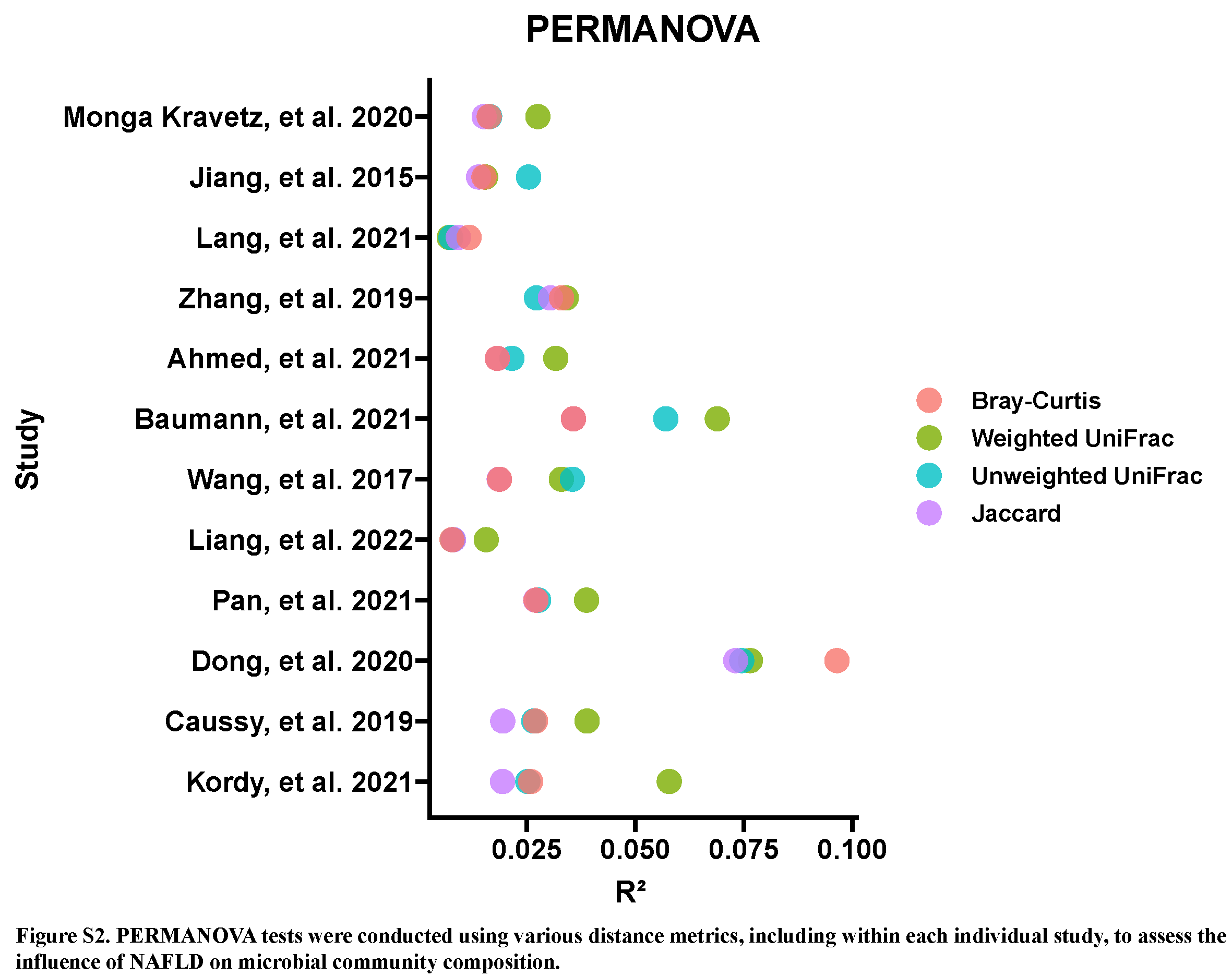

Supplement: Supplementary file 5 [file Image_2.tif]

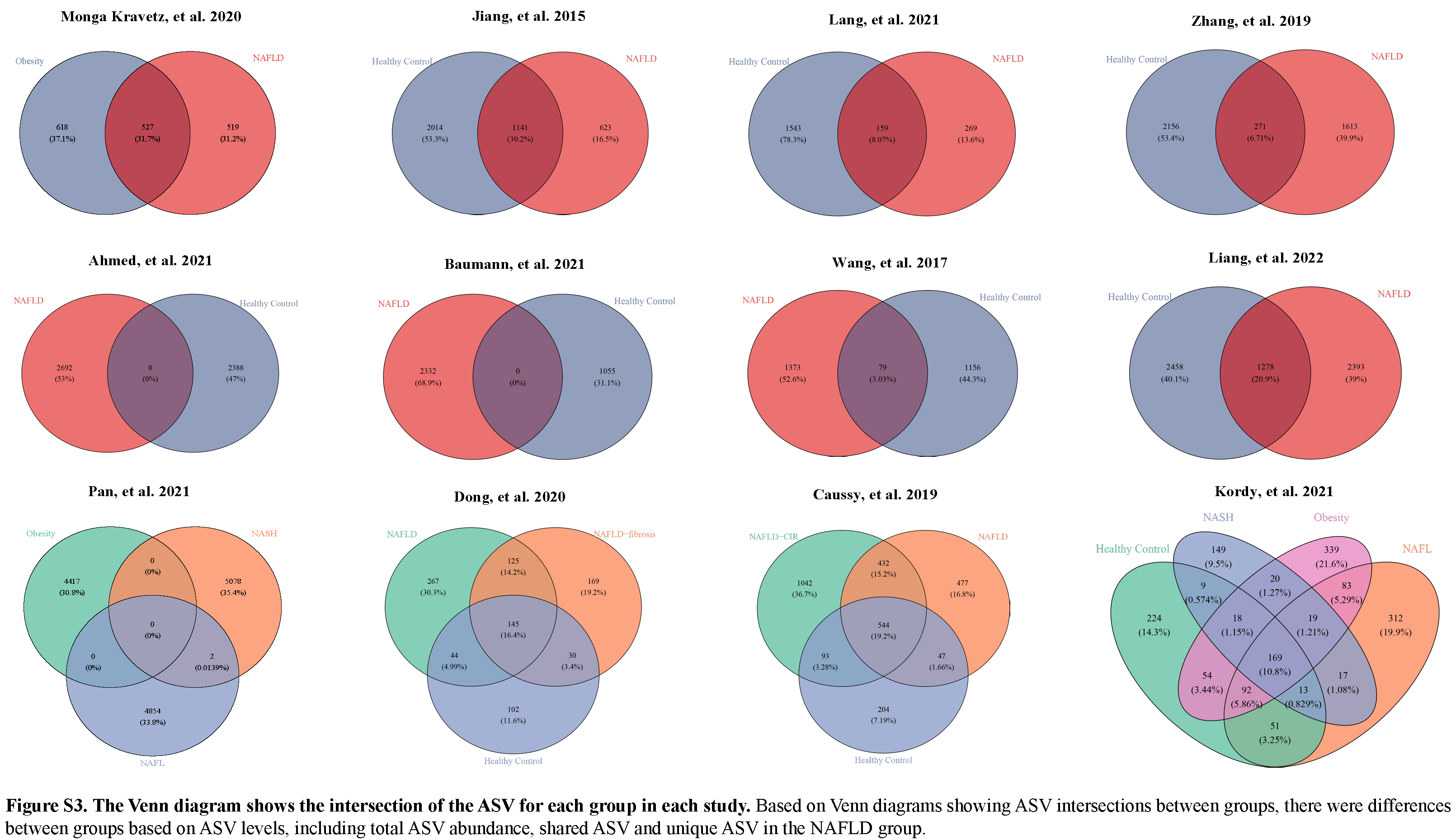

Supplement: Supplementary file 6 [file Image_3.tif]

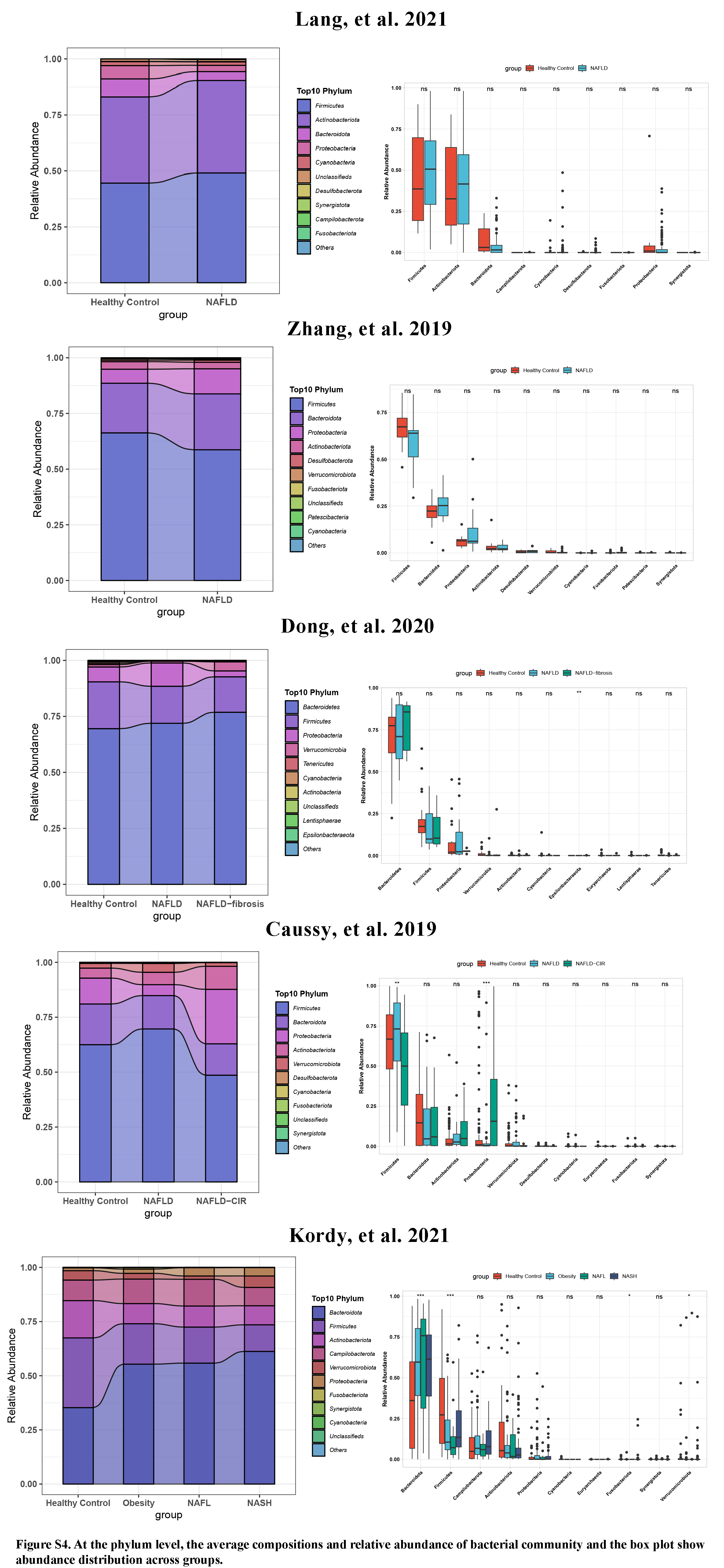

Supplement: Supplementary file 7 [file Image_4.tif]

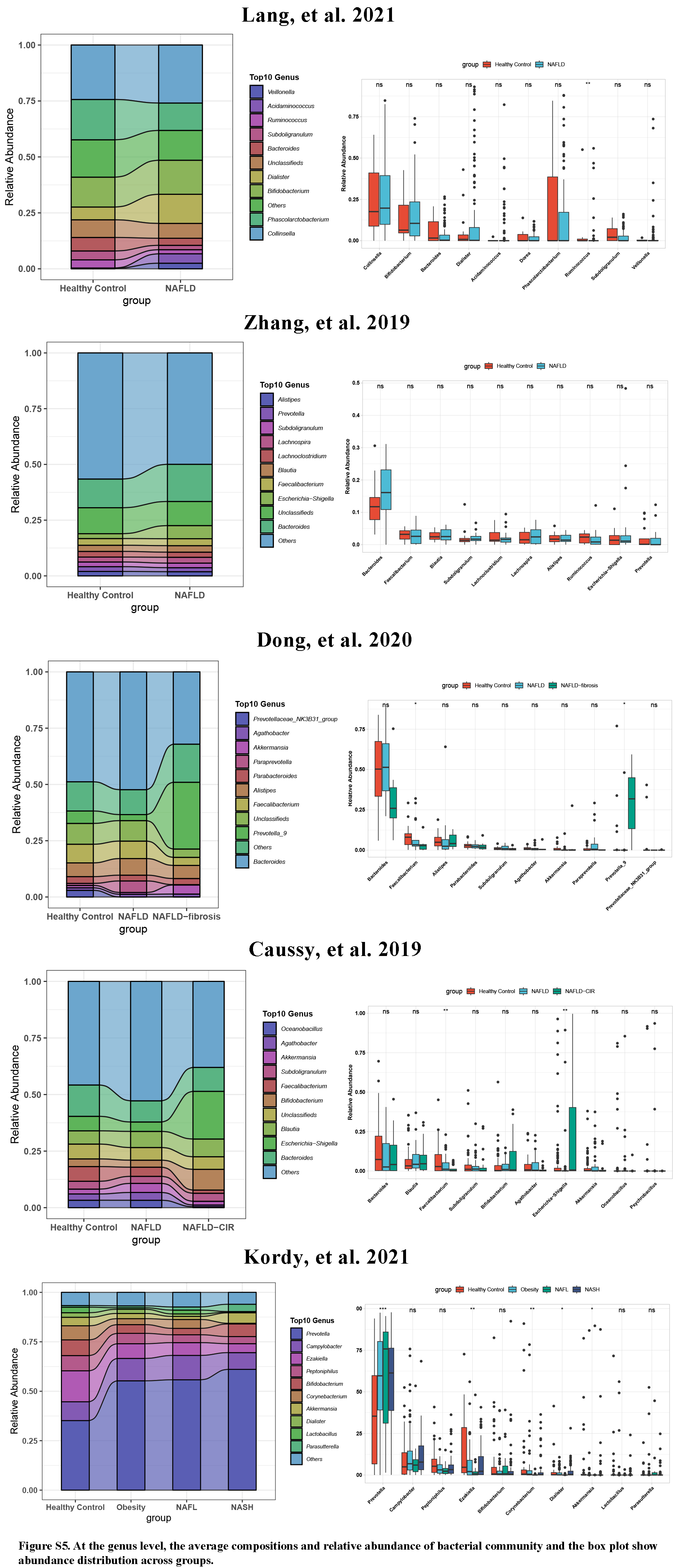

Supplement: Supplementary file 8 [file Image_5.tif]

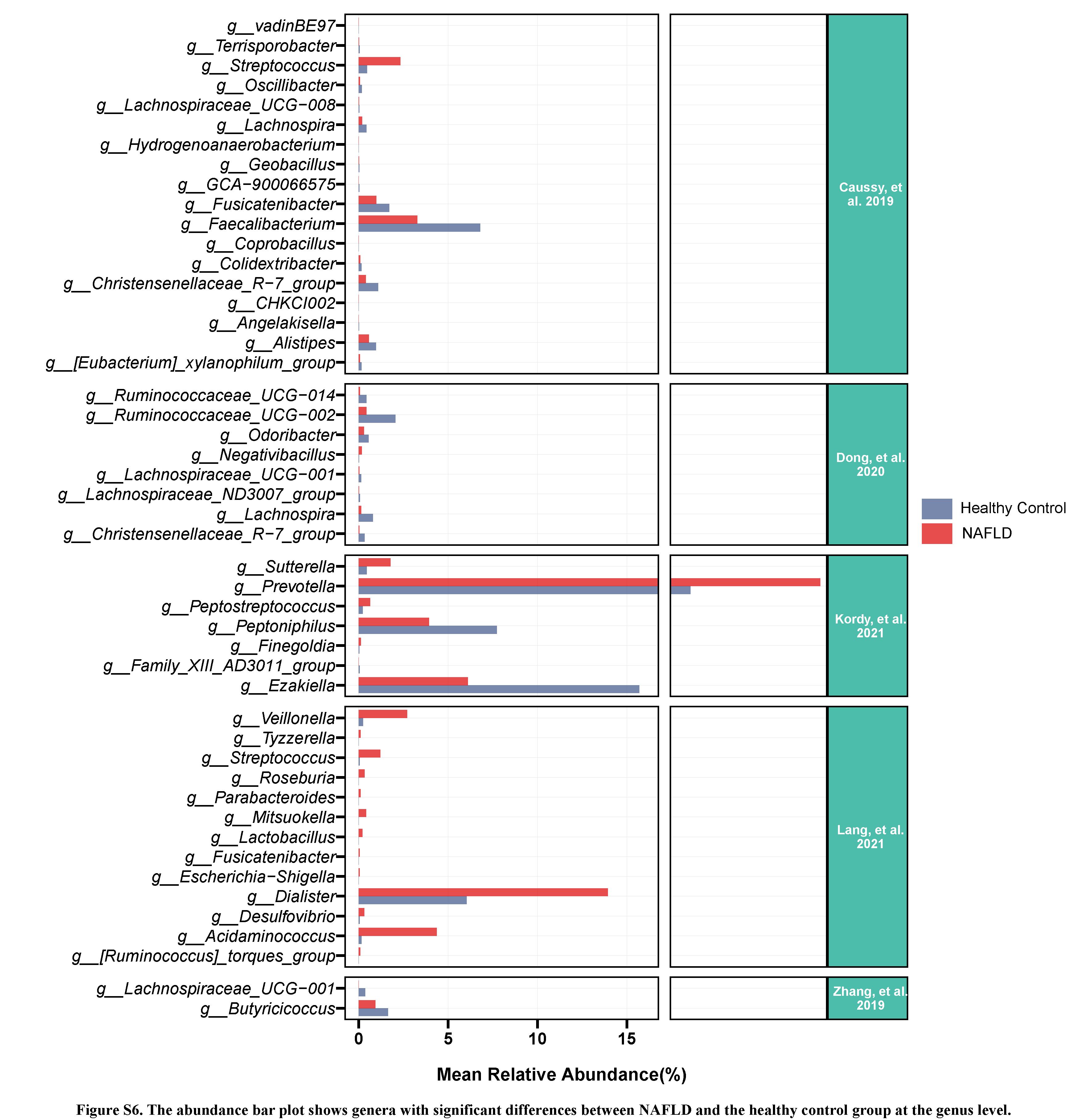

Supplement: Supplementary file 9 [file Image_6.tif]

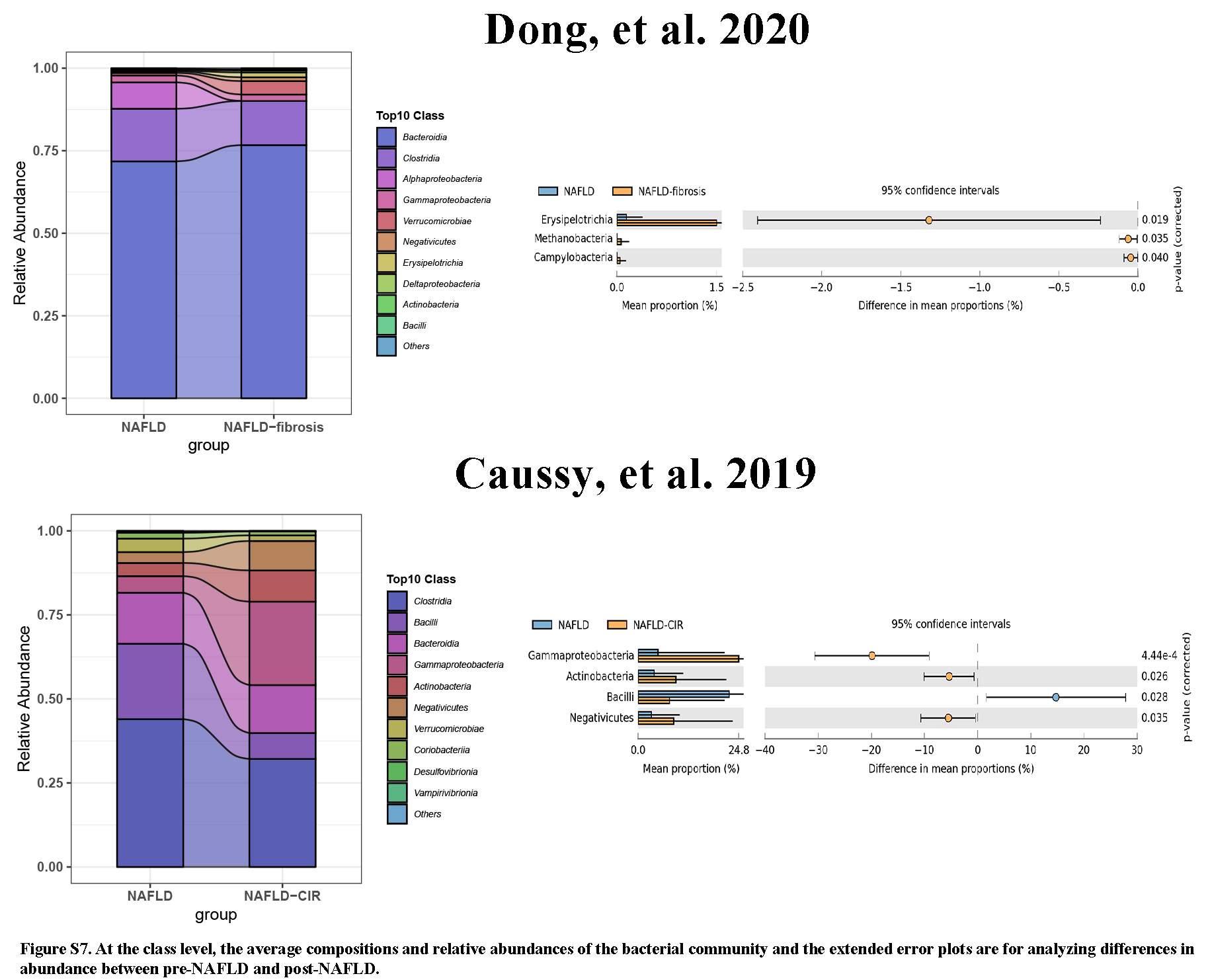

Supplement: Supplementary file 10 [file Image_7.tif]

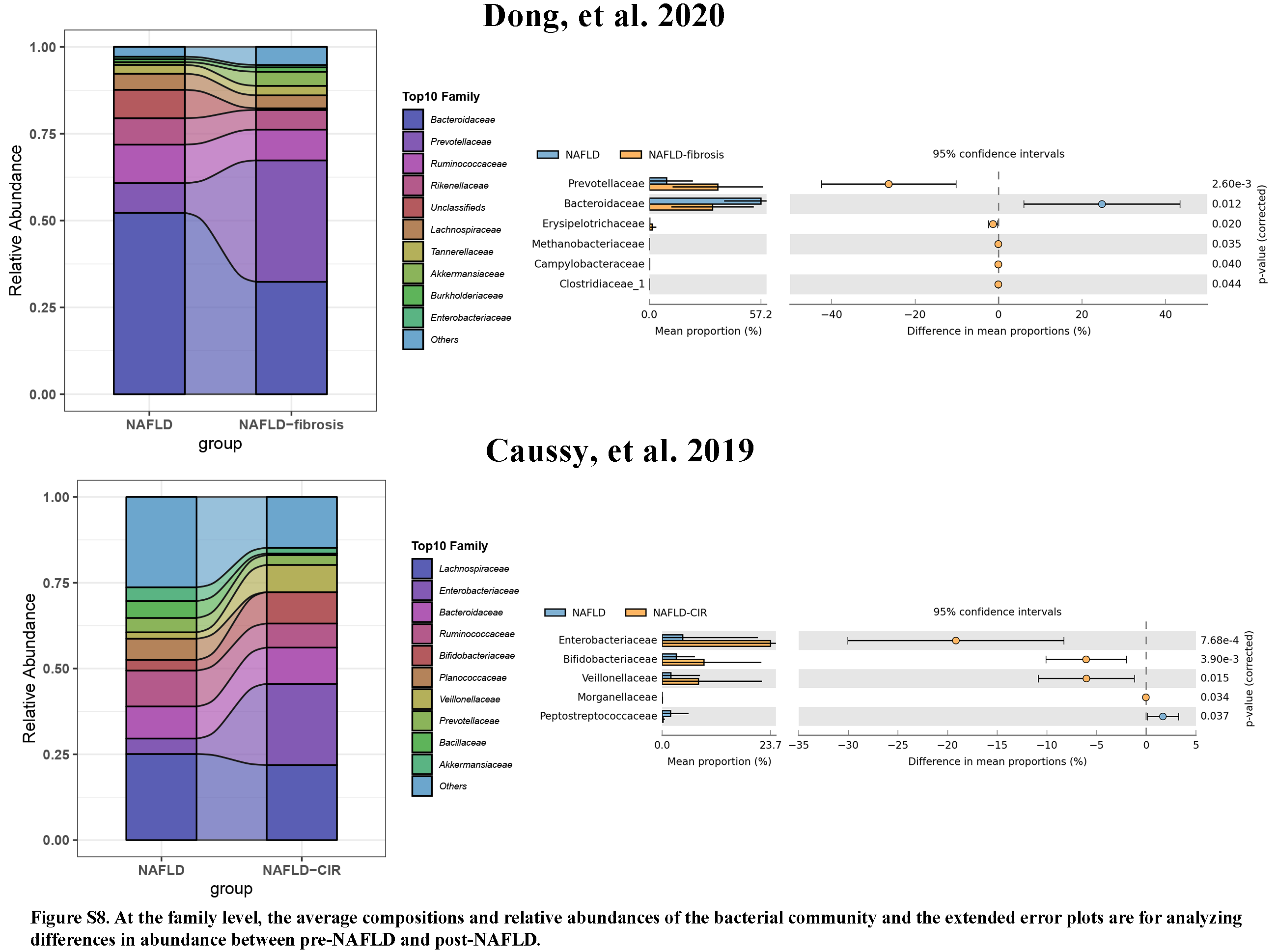

Supplement: Supplementary file 11 [file Image_8.tif]

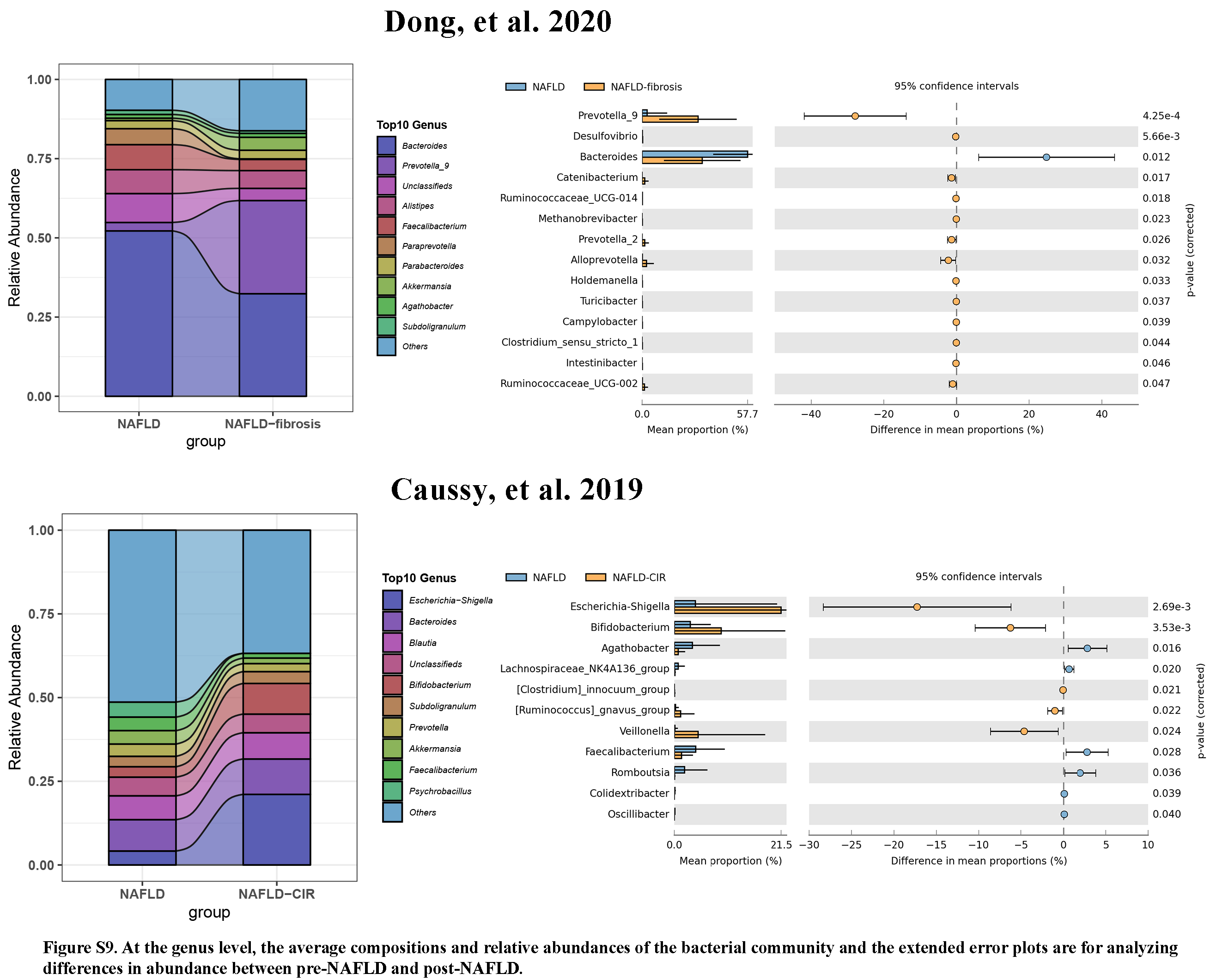

Supplement: Supplementary file 12 [file Image_9.tif]

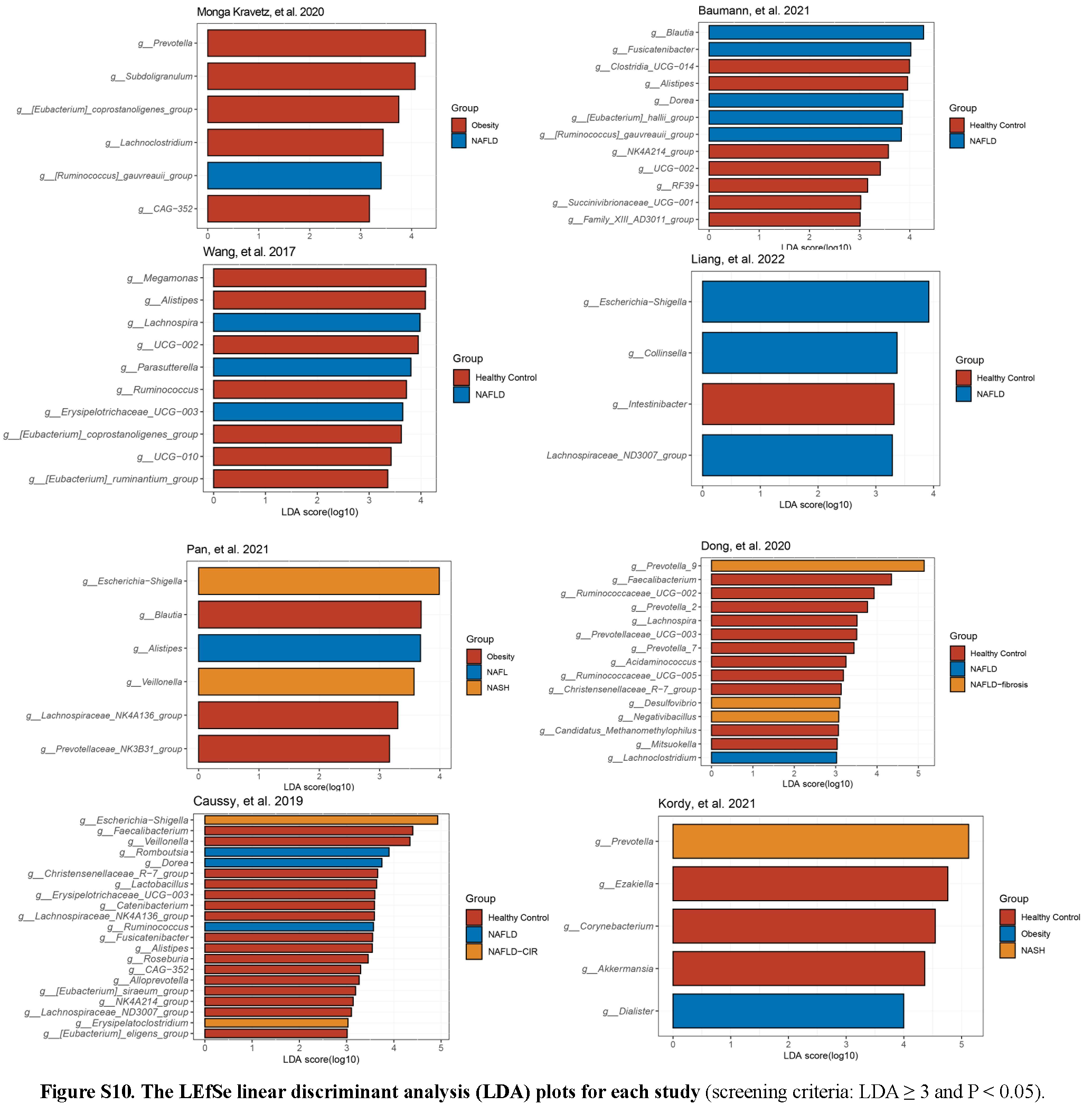

Supplement: Supplementary file 13 [file Image_10.tif]

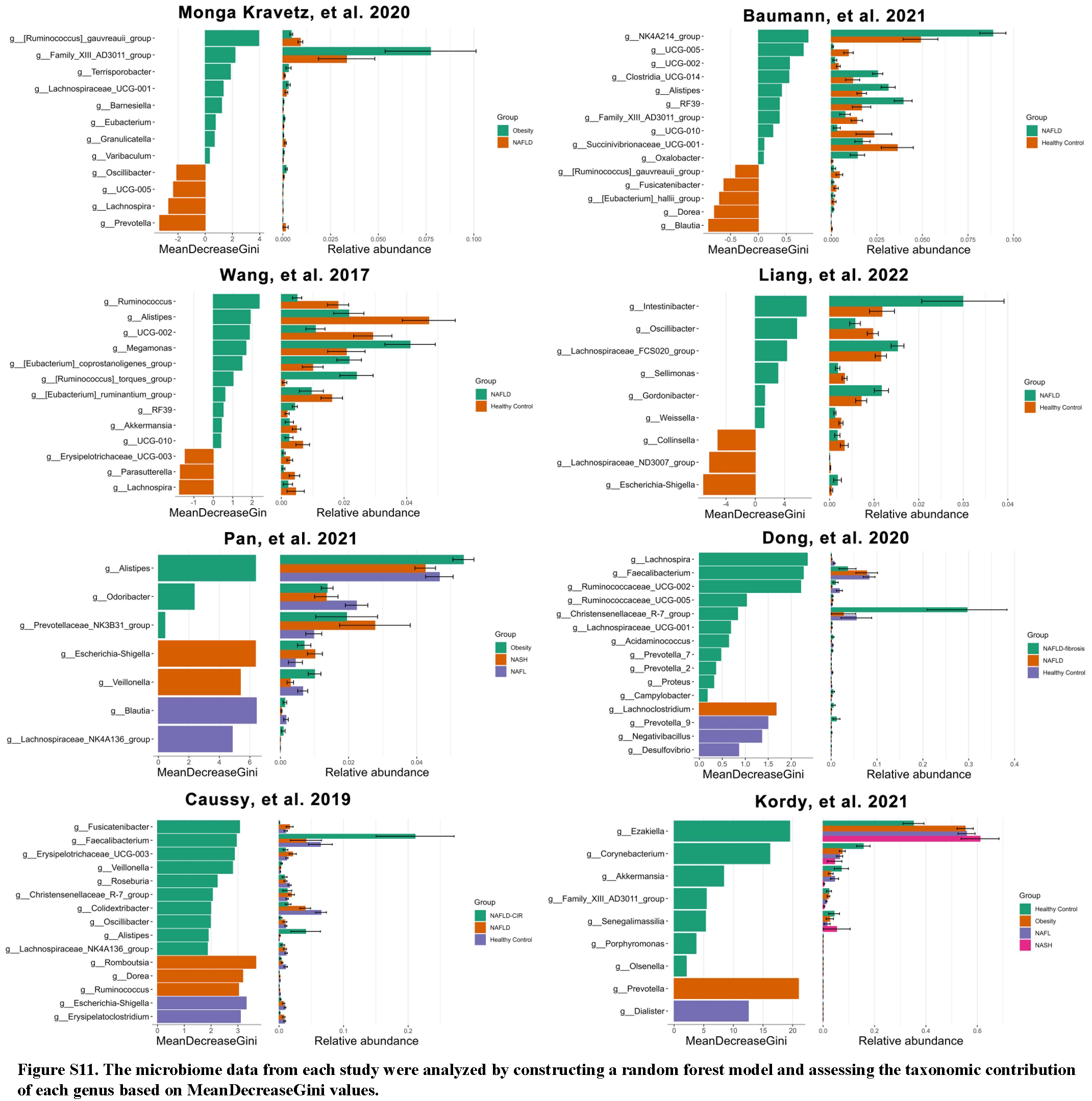

Supplement: Supplementary file 14 [file Image_11.tif]
